# Glucuronidation of deoxynivalenol (DON) by different animal species: identification of iso-DON glucuronides and iso-deepoxy-DON glucuronides as novel DON metabolites in pigs, rats, mice, and cows

**DOI:** 10.1007/s00204-017-2012-z

**Published:** 2017-06-21

**Authors:** Heidi E. Schwartz-Zimmermann, Christian Hametner, Veronika Nagl, Iris Fiby, Lukas Macheiner, Janine Winkler, Sven Dänicke, Erica Clark, James J. Pestka, Franz Berthiller

**Affiliations:** 1 Christian Doppler Laboratory for Mycotoxin Metabolism, Department of Agrobiotechnology (IFA-Tulln), Center for Analytical Chemistry, University of Natural Resources and Life Sciences, Vienna (BOKU), Konrad-Lorenz-Str. 20, 3430 Tulln, Austria; 2 Institute of Applied Synthetic Chemistry, Vienna University of Technology, Getreidemarkt 9/163-OC, 1060 Vienna, Austria; 3 BIOMIN Research Center, Technopark 1, 3430 Tulln, Austria; 4 Institute of Animal Nutrition, Friedrich-Loeffler-Institute (FLI), Federal Research Institute for Animal Health, 38116 Brunswick, Germany; 5 Department of Food Science and Human Nutrition, Michigan State University, East Lansing, MI 48824 USA

**Keywords:** Metabolism, Iso-deoxynivalenol, Iso-deepoxy-deoxynivalenol, Cyclic hemiketal DON 8-O-β-D-glucuronide, High-performance liquid chromatography–tandem mass spectrometry (HPLC–MS/MS)

## Abstract

The *Fusarium* mycotoxin deoxynivalenol (DON) is a frequent contaminant of cereal-based food and feed. Mammals metabolize DON by conjugation to glucuronic acid (GlcAc), the extent and regioselectivity of which is species-dependent. So far, only DON-3-glucuronide (DON-3-GlcAc) and DON-15-GlcAc have been unequivocally identified as mammalian DON glucuronides, and DON-7-GlcAc has been proposed as further DON metabolite. In the present work, qualitative HPLC–MS/MS analysis of urine samples of animals treated with DON (rats: 2 mg/kg bw, single bolus, gavage; mice: 1 mg/kg bw, single i.p. injection; pigs: 74 µg/kg bw, single bolus, gavage; cows: 5.2 mg DON/kg dry mass, oral for 13 weeks) revealed additional DON and deepoxy-DON (DOM) glucuronides. To elucidate their structures, DON and DOM were incubated with human (HLM) and rat liver microsomes (RLM). Besides the expected DON/DOM-3- and 15-GlcAc, minor amounts of four DON- and four DOM glucuronides were formed. Isolation and enzymatic hydrolysis of four of these compounds yielded iso-DON and iso-DOM, the identities of which were eventually confirmed by NMR. Incubation of iso-DON and iso-DOM with RLM and HLM yielded two main glucuronides for each parent compound, which were isolated and identified as iso-DON/DOM-3-GlcAc and iso-DON/DOM-8-GlcAc by NMR. Iso-DON-3-GlcAc, most likely misidentified as DON-7-GlcAc in the literature, proved to be a major DON metabolite in rats and a minor metabolite in pigs. In addition, iso-DON-8-GlcAc turned out to be one of the major DON metabolites in mice. DOM-3-GlcAc was the dominant DON metabolite in urine of cows and an important DON metabolite in rat urine. Iso-DOM-3-GlcAc was detected in urine of DON-treated rats and cows. Finally, DON-8,15-hemiketal-8-glucuronide, a previously described by-product of DON-3-GlcAc production by RLM, was identified in urine of DON-exposed mice and rats. The discovery of several novel DON-derived glucuronides in animal urine requires adaptation of the currently used methods for DON-biomarker analysis.

**Electronic supplementary material:**

The online version of this article (doi:10.1007/s00204-017-2012-z) contains supplementary material, which is available to authorized users.

## Introduction

Formed pre-harvest by *Fusarium* species, the mycotoxin deoxynivalenol (DON) is one of the most frequent fungal contaminants of food and feed worldwide. DON affects eukaryotic cells by inhibition of protein-, DNA-, and RNA synthesis, resulting in toxic effects in animals and plants (Rocha et al. 2005). These effects include feed refusal and emesis, growth retardation, and modulation of immune response in animals (Pestka 2010). However, plants and animals are capable of mitigating DON by conjugation. By attaching glycoside residues, plants can produce masked DON compounds like DON-3-glucoside (Berthiller et al. 2005, 2009). Depending on the animal species, animals conjugate DON to glucuronic acid (GlcAc) (reviewed by Payros et al. 2016) or sulfate (Schwartz-Zimmermann et al. 2015; Wan et al. 2014), which are both phase II metabolism reactions. Glucuronidation is the major conjugation reaction of DON in mammals, whereas sulfation is the dominant metabolization in poultry. Sulfonation has additionally been described for rats (Schwartz-Zimmermann et al. 2014; Wan et al. 2014), but its mechanism has not yet been elucidated. In addition to animal-innate phase II conjugation, DON can also be metabolized by gut microbes. The most prominent microbial metabolite of DON is deepoxy-DON (DOM)(Fuchs et al. 2002) which can, in turn, be subject to glucuronidation (Nagl et al. 2014), sulfation (Schwartz-Zimmermann et al. 2015), or sulfonation (Schwartz-Zimmermann et al. 2014).

**Fig. 1:**
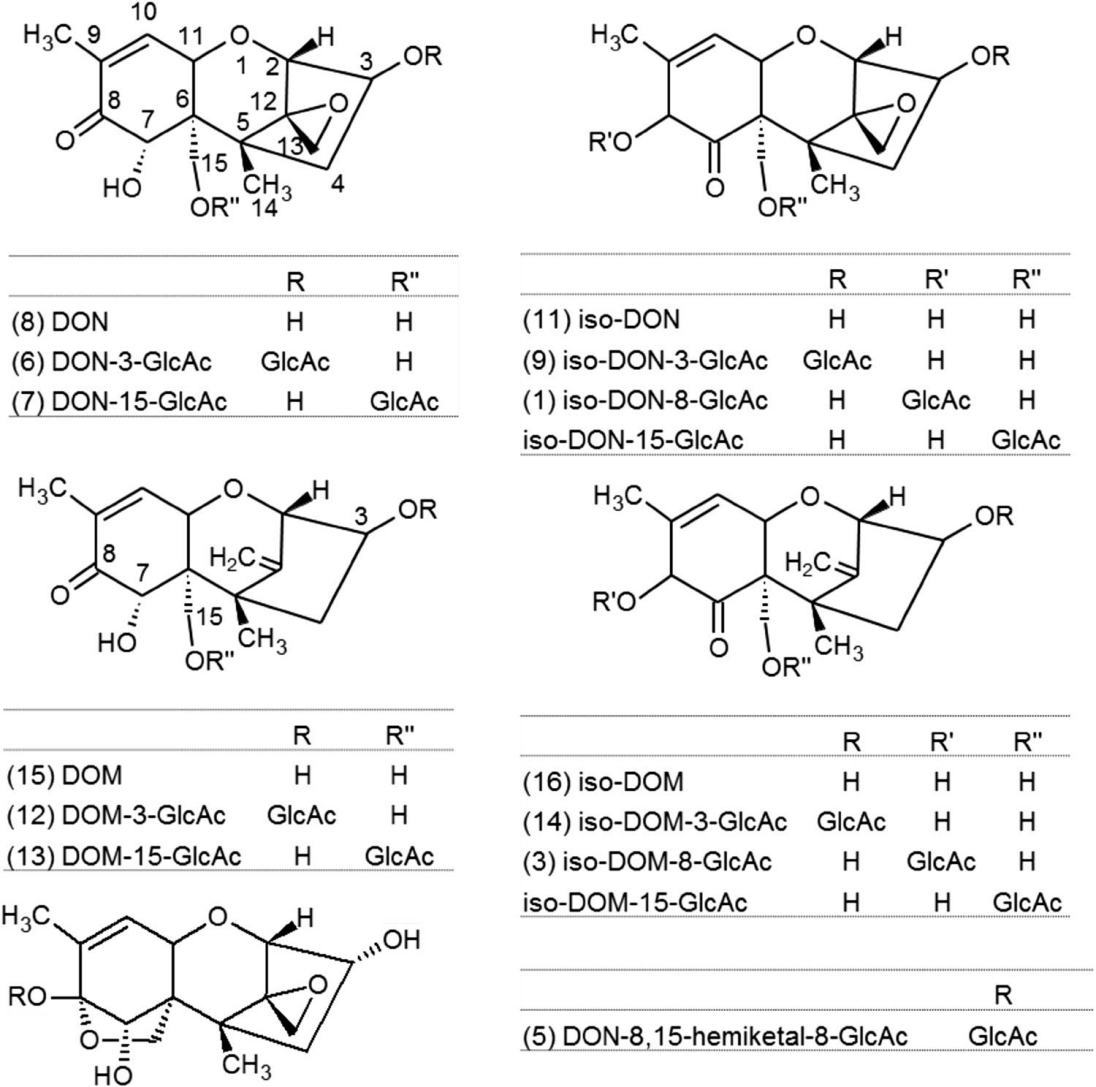
Chemical structures of DON, iso-DON, DOM, iso-DOM, and their glucuronides. In *brackets* compound numbers as in Fig. 2

The glucuronidation activities and the regiospecificity of glucuronidation towards DON are species-dependent (Maul et al. 2012, 2015; Uhlig et al. 2013). Pioneer work on in vitro DON glucuronidation by human liver microsomes (HLM) and liver microsomes of different animal species revealed DON-3-glucuronide (DON-3-GlcAc) as major DON metabolite upon incubation of DON with animal liver microsomes. DON-15-glucuronide (DON-15-GlcAc) was the prevailing conjugate upon incubation of DON with HLM (Maul et al. 2012, 2015), and was also readily formed by porcine liver microsomes (Maul et al. 2015). In addition, a third DON-GlcAc was detected, which was formed in considerable amounts by rat-, bovine-, trout-, and carp liver microsomes and tentatively identified as DON-7-glucuronide (DON-7-GlcAc). Finally, a cyclic DON-8,15-hemiketal-8-GlcAc could be isolated as a side product formed upon incubation of DON with Wistar rat liver microsomes and structurally elucidated by nuclear magnetic resonance spectroscopy (NMR) (Uhlig et al. 2013, 2016). Chemical structures of DON-3-GlcAc, DON-15-GlcAc, and DON-8,15-hemiketal-8-GlcAc are given in Fig. 1.

In humans, DON-15-GlcAc is formed preferentially over DON-3-GlcAc (Sarkanj et al. 2013; Warth et al. 2012, 2013). A third DON-GlcAc that was tentatively identified as DON-7-GlcAc was additionally detected in some highly contaminated human urine samples (Sarkanj et al. 2013; Warth et al. 2013). In pigs orally treated with DON, formation of DON-3-GlcAc and DON-15-GlcAc at similar ratio was observed, albeit with notable differences between individual animals (Nagl et al. 2014). In rats, DON-3-GlcAc prevailed, but the presence of a minor second DON-GlcAc peak that was not DON-15-GlcAc was detected (Nagl et al. 2012; Veršilovskis et al. 2012). In addition, enzymatic hydrolysis of urine samples collected from pigs treated with DON-3-glucoside indicated formation of DOM glucuronides (Nagl et al. 2014). Summarizing the literature data, only DON-3-GlcAc and DON-15-GlcAc were unequivocally identified in urine samples of humans and animals so far. Formation of a third DON-GlcAc has been reported in human and rat urine, but confirmation of the suggested structure (DON-7-GlcAc) is still outstanding. A forth glucuronide produced by rat liver microsomes has been structurally elucidated as DON-8,15-hemiketal-8-GlcAc, but never been detected in animal urine. Finally, formation of DOM glucuronides has been suggested, but not confirmed.

The main aim of our work was to investigate in vivo DON glucuronidation by different animal species. To this end, we analyzed urine samples of DON-treated rats, mice, pigs, and cows by a flat gradient HPLC–MS/MS method. We then converted DON and DOM with rat- and human liver microsomes, collected the main and minor glucuronides, produced greater amounts of the relevant novel glucuronides, and elucidated their structures. The discovery and identification of several novel DON- and DOM glucuronide metabolites formed by different animal species are of great importance with respect to inter-species variation in DON metabolism, toxicology, and analytical determination of DON metabolites as biomarker of DON exposure.

## Materials and methods

### Reagents and standards

Acetonitrile (ACN, gradient grade) was purchased from VWR International GmbH (Vienna, Austria). Acetic acid (LC–MS gradient grade), sodium methoxide solution (25% w/v in methanol), β-glucuronidase (*Escherichia coli*, Type IX-A, 675 kU/g solid), alamethicin, magnesium chloride, uridine-diphosphoglucuronic acid (UDPGA), uridine-diphospho-*N*-acetylglucosamine (UDPAG), and creatinine were obtained from Sigma Aldrich (Vienna, Austria). Formic acid (98–100%, p.a.) and glacial acetic acid (100%) for semi-preparative chromatography were provided by Merck (Darmstadt, Germany) and water-free methanol was purchased from Roth (Karlsruhe, Germany). Rat liver microsomes (RLM, pooled liver microsomes from 25 male Sprague–Dawley rats, 20 mg/mL) and human liver microsomes (HLM, pooled liver microsomes from 25 donors, and mixed gender, 20 mg/mL) were delivered by BioreclamationIVT (Brussels, Belgium). All reagents for preparation of buffer solutions [TRIS, phosphate buffered saline (PBS)] were p.a. grade. In all experiments, ultra-pure water (purified by a Purelab Ultra system ELGA LabWater, Celle, Germany) was used.

Solid deoxynivalenol (DON, purity >95%) as well as liquid calibrant solutions of DON and DOM (100 and 50 mg/L, respectively, in ACN) were supplied by Romer Labs GmbH (Tulln, Austria). DOM as the starting material for glucuronide production was obtained by conversion of DON with the bacterial strain BBSH 797 as described in Schwartz-Zimmermann et al. (2014) and purified by preparative chromatography as outlined in Schwartz-Zimmermann et al. (2015). DON-3-GlcAc produced by chemical synthesis (Fruhmann et al. 2012) and dissolved in MeOH to a concentration of 10 mg/L served as reference standard. DON-8,15-hemiketal-8-GlcAc was provided by Silvio Uhlig (Norwegian Veterinary Institute, Oslo) and used for compound identification.

### Microsome assay for glucuronidation of DON and DOM

The assay for glucuronidation of DON and DOM was based on the protocol published by Uhlig et al. (2013). For small-scale production of DON- and DOM glucuronides, pre-mixes containing all components required for the reaction except the microsomes were prepared. The pre-mixes (described for 5 replicates) were composed of 100 µL each of UDPGA (100 mM), UDPAG (5 mM), alamethicin (250 µg/mL in ethanol/water 5/100, v/v), MgCl_2_ (50 mM), Tris–HCl (1 M), and aqueous mycotoxin solution (4000 mg/L for DON and DOM) as well as 350 µL of water. Prior to the addition of microsomes, 190 µL aliquots of the pre-mixes were pipetted into Eppendorf reaction vials and pre-incubated at 37 °C for 10 min. Subsequently, 10 µL of RLM or HLM was added and the tubes were incubated under slight shaking at 37 °C overnight. The reactions were stopped by the addition of 800 µL of cold MeOH and proteins were removed by centrifugation at 14,000×*g* for 10 min. In total, five replicates were prepared for each toxin/microsome combination and the combined supernatants after centrifugation were concentrated to 0.3 mL by evaporation. Prior to semi-preparative chromatography, aliquots of the reaction mixtures were checked by HPLC–MS/MS (see below). The relative abundances of the formed glucuronides were estimated based on the peak areas of the glucuronide specific selected reaction monitoring transitions 471.1–>113.0 (DON-GlcAc) and 455.1–>113.0 (DOM-GlcAc).

### Semi-preparative isolation of glucuronides produced in the microsome assays

Isolation of the reaction products formed upon incubation of DON and DOM with rat and human liver microsomes was carried out by semi-preparative chromatography on an Agilent 1100 series preparative HPLC system (Agilent Technologies, Waldbronn, Germany). Compounds were separated by gradient elution on a Kinetex C18 column (150 × 10 mm, 5 μm, Phenomenex, Aschaffenburg, Germany) with a pre-column of the same material using water and ACN, both containing 0.1% acetic acid, as mobile phases A and B. The flow rate was 6 mL/min, the column temperature 25 °C, and the injection volume was 300 µL. Gradient elution started with an isocratic period of 0.5 min at 6% B and continued with a linear increase to 13.8% B within further 7.5 min. Subsequently, the proportion of B was increased to 90% within 0.5 min and the column was flushed until 10.5 min. Finally, the column was re-equilibrated at 6% B for 2 min. Forty fractions were collected at equal time intervals (12 s/fraction) between 2.5 and 10.5 min. Compounds were detected by UV-detection at 200, 254, and 280 nm. All collected fractions were analyzed by HPLC–MS/MS (see below) for the presence of DON- and DOM glucuronides.

### Enzymatic hydrolysis

5 µL aliquots of stock solutions containing ca. 5 mg/L of each isolated (iso-)DON/DOM glucuronide were evaporated and incubated in 50 µL of 40 mM PBS containing 650 U of β-glucuronidase at 37 °C over night. Prior to LC–MS/MS analysis, proteins were removed by the addition of 150 µL of MeOH and centrifugation.

### Production and purification of iso-DON and iso-DOM

For production of iso-DON, 30 mg of solid DON was dissolved in 10 mL of absolute methanol, and 20 µL of sodium methoxide solution (25% w/v in methanol) was added. The solution was shaken at ambient temperature for 20 h. The progress of the reaction was monitored by analyzing diluted aliquots by LC–MS/MS and LC–UV. The reaction was stopped by the addition of 10 mL water/formic acid (99/1, v/v). Prior to purification by semi-preparative chromatography, the volume of the solution was reduced to 4 mL on a rotary evaporator. As the yield of iso-DON was only between 5 and 10%, the procedure was repeated twice with the DON regained upon semi-preparative isolation.

Iso-DOM was discovered to be a side product of the DOM production by conversion of DON by the anaerobic bacterial strain BBSH 797. Upon preparative isolation of DOM according to Schwartz-Zimmermann et al. (2015), both a pure fraction of DOM (5.66–6.03 min) and a mixed fraction containing DOM and iso-DOM (6.05–6.40 min) were collected. The mixed fraction was subjected to semi-preparative chromatography for separation of DOM and iso-DOM.

Purification of iso-DON and iso-DOM was carried out by semi-preparative chromatography using the same conditions as described above for isolation of glucuronides produced in the microsome assay. The gradient for purification of iso-DON was the same as described for isolation of glucuronides (see above). Residual DON was collected between 6.40 and 6.95 min, pure iso-DON was collected between 7.15 and 7.45 min, and two fractions containing iso-DON and either one earlier or one later eluting side product were collected between 6.96 and 7.14 min and between 7.46 and 7.65 min. Separation of DOM and iso-DOM started at 8% B for 0.5 min, continued with a linear increase to 17% B until 9 min and a steep increase to 90% which was reached at 10 min. The column was flushed at 90% B for 1 min and re-equilibrated at 8% B until 13 min. DOM was collected between 7.60 and 8.10 min and iso-DOM between 8.20 and 8.60 min.

The two fractions containing iso-DON and one of the two side products mentioned above were subjected to HPLC separation on an Agilent 1290 series UHPLC system equipped with a programmable switching valve (VICI Valco Instruments, Houston, Texas, USA). The column was the same as used for HPLC–MS/MS analysis (see below). A flat gradient (0–0.5 min: 5% B, 0.5–7.5 min: linear increase to 13% B, 7.5–8 min: linear increase to 95% B, 8–9 min: 95% B, 9.1–12 min: 5% B) was used for separation, the injection volume was 10 µL, and iso-DON was collected between 6.6 and 6.9 min.

### Production and purification of DOM-, iso-DON-, and iso-DOM glucuronides

**Fig. 2:**
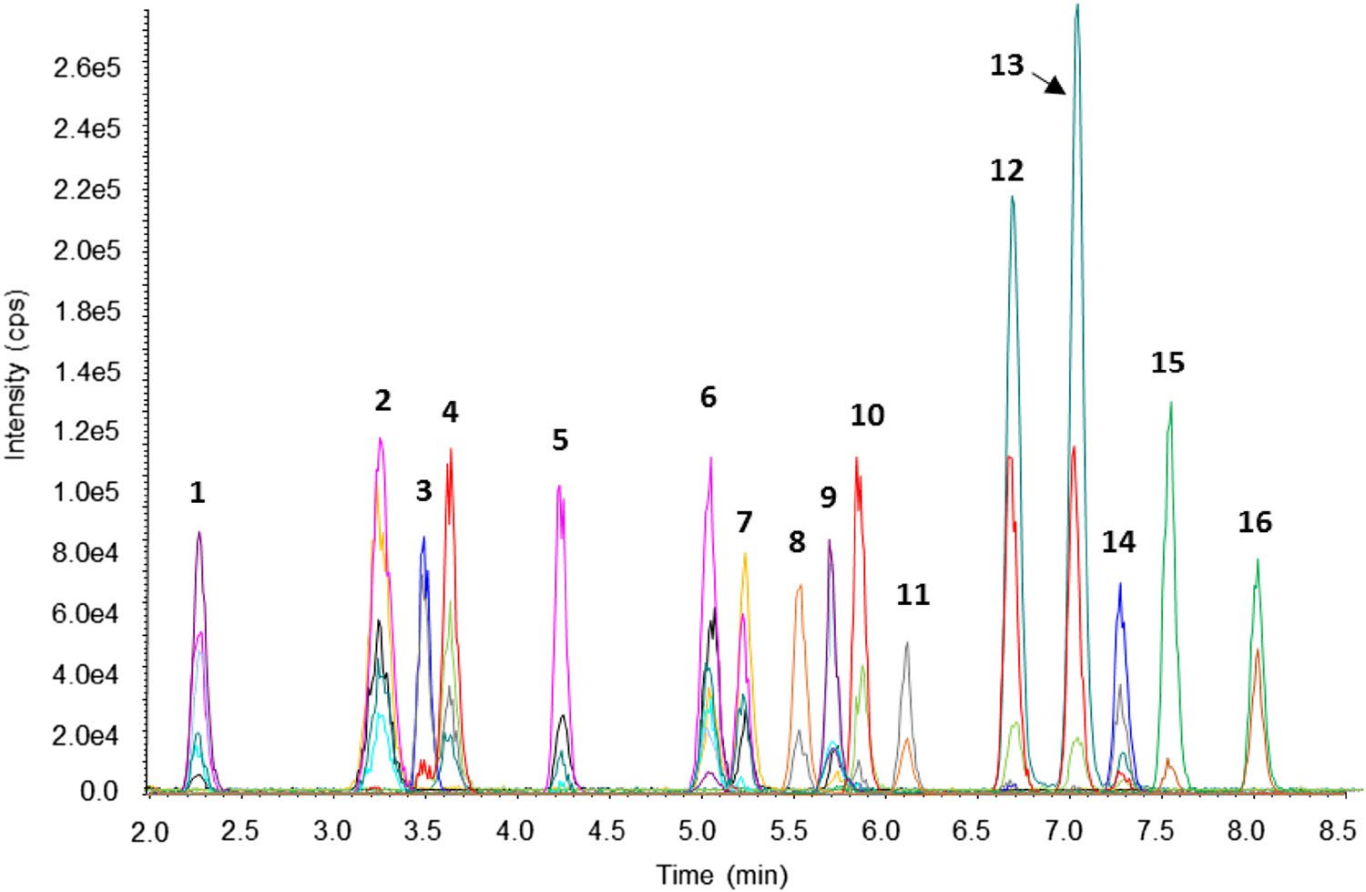
LC–MS/MS chromatogram of DON, iso-DON, DOM, iso-DOM, and all of their glucuronides isolated after incubation of DON and DOM with RLM and HLM. The concentration of the aglycones, DON- and DOM glucuronides was ca. 100 ng/mL, and the concentration of the iso-DON- and iso-DOM glucuronides was ca. 30 ng/mL. Annotation was based on NMR measurements (*1*,* 3*,* 9*,* 11*,* 12*,* 13*,* 14*, and* 16*), comparison with reference standards (*5*,* 6*,* 8*, and* 15*), and literature (7). *1* iso-DON-8-GlcAc, *2* unknown DON-GlcAc, *3* iso-DOM-8-GlcAc, *4* unknown DOM-GlcAc a, *5* DON-8,15-hemiketal-8-glucuronide, *6* DON-3-GlcAc, *7* DON-15-GlcAc, *8* DON, *9* iso-DON-3-GlcAc, *10* unknown DOM-GlcAc b, *11* iso-DON, *12* DOM-3-GlcAc, *13* DOM-15-GlcAc, *14* iso-DOM-3-GlcAc, *15* DOM, *16* iso-DOM

Comparison of glucuronides formed in the microsome assays with the glucuronide pattern in animal urine revealed compounds no. 1, 5, 6, 7, 9, 12, 13, and 14 (see Fig. 2) as the most relevant glucuronides. Of these, 5, 6, and 7 were identified as DON-8,15-hemiketal-8-GlcAc, DON-3-GlcAc, and DON-15-GlcAc. Therefore, our aim was to produce milligram-amounts of 1, 9, 12, 13, and 14 for consecutive structure elucidation by NMR. The parent compound of 1 and 9 was iso-DON, the aglycone of 12 and 13 was DOM, and the parent compound of 14 was iso-DOM. Production of DOM-, iso-DON-, and iso-DOM glucuronides was carried out by microsome assays as described above. Iso-DON and iso-DOM were incubated with RLM, whereas DOM was incubated with RLM for production of 12 and with HLM for production of 13. In total, between 10 and 30 replicates were prepared for each toxin/microsome combination to gain sufficient amounts. In addition, also iso-DON and iso-DOM were incubated with HLM at small scale to obtain the full glucuronidation pattern.

Purification of the formed glucuronides was carried out by semi-preparative chromatography as described above for the products of the first microsome assays. Four compounds were isolated from the reaction mixture of iso-DON with RLM: compound 1 (3.00–3.80 min), a novel compound eluting between 6.05 and 6.75 min, compound 9 (6.80–7.20 min), and residual iso-DON (7.21–7.50). Likewise, four compounds were obtained from the reaction mixture of iso-DOM with RLM: compound 3 (3.80–4.50 min), a novel compound eluting from 8.00–8.40 min, compound 14 (8.40–8.90 min), and non-converted iso-DOM (9.50–9.80 min). Incubation of DOM with RLM yielded compound 12 as major product, compound 13 as side product, and residual DOM. Compound 12 was isolated between 7.50 and 8.00 min and a mixed fraction containing 12 and 13 was collected between 8.00 and 8.3 min. Vice versa, incubation of DOM with HLM yielded compound 13 as main and compound 12 as side product. In that case, a mixed fraction was collected from 7.50–7.85 min and compound 13 was collected between 7.86 and 8.45 min. The mixed fractions were again subjected to semi-preparative chromatography. In both DOM reaction mixtures, non-converted DOM eluted between 8.60 and 9.10 min.

In the case of compounds 1 and 9, the resolution achievable by semi-preparative chromatography was not sufficient. Compound 1 partly co-eluted with a large reagent peak of slightly earlier retention time. Compound 9 partly co-eluted with iso-DON. Hence, the respective pooled fractions obtained by semi-preparative chromatography were up-concentrated and the analytes were isolated by HPLC on the same system as described for HPLC isolation of iso-DON. The column, mobile phases, and gradient used were the same as for HPLC–MS/MS analysis. The injection volume was increased to 10 µL for isolation. Compound 1 was collected between 2.1 and 2.4 min and compound 9 between 5.6 and 5.9 min.

### NMR

NMR spectra were obtained in methanol-d_4_ at 293 K on a Bruker Avance III HD spectrometer (Bruker BioSpin GmbH, Rheinstetten, Germany) equipped with a 5-mm Cryoprobe™ Prodigy BBO, operating at 600.15 MHz for ^1^H and 150.90 MHz for ^13^C. NMR data were recorded and processed using TopSpin 3.2 (Bruker BioSpin GmbH). Chemical shifts were established based on residual solvent signals (3.31 ppm for ^1^H and 49.15 ppm for ^13^C) and reported relative to TMS.

### HPLC–HR-MS and HPLC–HR-MS/MS analysis

High-performance liquid chromatography–high-resolution (tandem) mass spectrometry (HPLC–HR-MS/MS) analysis was performed on a 6550 iFunnel Q-TOF instrument coupled to a 1290 Infinity UHPLC system (both Agilent Technologies, Waldbronn, Germany). Chromatographic separation was achieved on a Kinetex C18 column (150 × 2.1 mm, 2.6 μm, Phenomenex) at a flow rate of 0.25 mL/min using a linear gradient (0–0.5 min: 5% B, 7–8 min: 100% B, 8.1–11 min: 5% B). Mobile phase A was water/formic acid (99.9/0.1, v/v) and mobile phase B MeOH/formic acid (99.9/0.1, v/v). Compounds were deprotonated by electrospray ionization (ESI) in the negative mode and measured first in full scan (*m/z* 100–500) and then in targeted MS/MS mode (*m/z* 40–500) at different collision energies (CEs) between −20 and −40 eV. ESI was carried out at a gas temperature of 130 °C, drying gas flow of 16 L/min, nebulizer pressure of 30 psig, sheath gas temperature of 300 °C, and sheath gas flow of 11 L/min. The capillary voltage was 4000 V and the nozzle voltage 500 V. Data acquisition was achieved in the 2 GHz extended dynamic range mode and the acquisition rate was set to 333 ms/spectrum.

### HPLC–MS/MS analysis

High-performance liquid chromatography–tandem mass spectrometry (HPLC–MS/MS) analyses were performed on an Agilent 1290 series UHPLC system coupled to a 6500+ QTrap mass spectrometer equipped with an IonDrive Turbo V^®^ ESI source (both Sciex, Foster City, CA, USA). Analytes were separated on a Kinetex C18 column (150 × 2.1 mm, 2.6 μm) protected by a SecurityGuard ULTRA pre-column of the same stationary phase (both Phenomenex, Aschaffenburg, Germany) at 30 °C and at a flow rate of 0.25 mL/min. Mobile phases A and B consisted of water/acetic acid and ACN/acetic acid (both 99.9/0.1, v/v), respectively. The gradient started with an isocratic period at 5% B for 0.5 min and continued with a linear increase to 15% B until 7 min, followed by a further linear increase to 30% B between 7 and 8.5 min and a steep increase to 100% B until 9 min. Finally, the column was washed at 100% B for 1.5 min and re-equilibrated at 5% B until 13.5 min. The injection volume was 3 μL and the LC eluent was diverted to the MS between 2.0 and 9.5 min.

Tandem mass spectrometric detection was performed in selected reaction monitoring (SRM) mode after ESI in negative polarity. The ion source settings were: temperature 400 °C, ion spray voltage −4500 V, curtain gas 35 psi, ion source gas 1 60 psi, ion source gas 2 40 psi, and collision gas (N_2_) high. First measurements of animal urine samples and of fractions obtained by semi-preparative chromatography after incubation of DON and DOM with RLM and HLM were carried out with four SRM transitions optimized for DON-3-GlcAc and four transitions calculated for DOM glucuronides. For detection of DON glucuronides, the deprotonated precursor ion (*m/z* 471.1) was fragmented to two glucuronide-derived fragments (*m/z* 113.0, collision energy (CE) −35 eV; *m/z* 175.1, CE −40 eV), to one DON-specific fragment (*m/z* 265.1, CE −38 eV), and to one fragment formed by loss of the CH_2_O group attached at C-6 (see Fig. 1) which distinguishes DON-3-GlcAc from DON-15-GlcAc (*m/z* 441.1, CE −30 eV). For detection of DOM glucuronides, the deprotonated precursor (*m/z* 455.1) was fragmented to the corresponding fragment ions (*m/z* 113, 175, 249, 425), using the same CEs as for the respective DON-GlcAc fragment ions. SRM transitions for DON and DOM, optimized by software controlled compound optimization, are listed in Table 1. The final SRM method was established by combining the SRM transitions optimized for the individual isolated glucuronides (see Table 1). Analyst^®^ software version 1.6.3 (Sciex) was used for instrument control and data evaluation.

**Table 1:** Selected reaction monitoring transitions

Analyte	Retention time (min)	Precursor ion (*m/z*)	Ion species	Product ions (quant/qual1/qual2) (*m/z*)	CE (eV)	Rel. intensity (qual1/quant)/(qual2/quant)
(1) Iso-DON-8-GlcAc	2.22	471.1	[M–H]^−^	441.1/113.0	−25/−35	0.54
(2) Unknown DON-GlcAc	3.26	471.1	[M–H]^−^	113.0/193.1	−35/−30	0.97
(3) Iso-DOM-8-GlcAc	3.49	455.1	[M–H]^−^	425.1/249.1	−25/−36	0.88
(4) Unknown DOM-GlcAc a	3.62	455.1	[M–H]^−^	113.0/175.1	−35/−30	0.61
(5) DON-8,15-hemiketal-8-GlcAc	4.23	471.1	[M–H]^−^	113.0/175.1	−35/−40	0.29
Iso-DON-15-GlcAc	4.92	471.1	[M–H]^−^	193.1/113.0	−30/−35	0.19
(6) DON-3-GlcAc	5.03	471.1	[M–H]^−^	113.0/175.1/441.1	−35/−40/−30	0.66/0.21
(7) DON-15-GlcAc	5.23	471.1	[M–H]^−^	193.1/113.0	−30/−35	0.72
(8) DON	5.56	355.1	[M+ CH_3_COO]^−^	59.0/265.1	−38/−18	0.24
(9) Iso-DON-3-GlcAc	5.71	471.1	[M–H]^−^	441.1/229.0	−25/−43	0.24
(10) Unknown DOM-GlcAc b	5.84	455.1	[M–H]^−^	113.0/175.1	−35/−30	0.39
(11) Iso-DON	6.13	355.1	[M+ CH_3_COO]^−^	265.1/59.0	−18/−38	0.42
(12) DOM-3-GlcAc	6.65	455.1	[M–H]^−^	193.1/113.0/425.1	−30/−35/−25	0.51/0.01
(13) DOM-15-GlcAc	7.01	455.1	[M–H]^−^	193.1/113.0	−30/−35	0.34
iso-DOM-15-GlcAc	7.03	455.1	[M–H]^−^	193.1/113.0	−30/−35	0.24
(14) Iso-DOM-3-GlcAc	7.26	455.1	[M–H]^−^	425.1/249.1	−25/−36	0.53
(15) DOM	7.57	325.1	[M+ CH_3_COO]^−^	59.0/249.1	−40/−17	0.08
(16) Iso-DOM	8.05	325.1	[M+ CH_3_COO]^−^	59.0/249.1	−40/−17	0.63

The number in brackets corresponds to the number of the compound in Fig. 2

*quant* quantifier, *qual* qualifier, *CE* collision energy

### Animal trials

For studying the glucuronidation of DON in different animal species, urine samples collected in the course of several previous animal experiments (*n* = 4 per for each trial) were diluted to 0.5 mM creatinine and re-analyzed by the HPLC–MS/MS method described above. A brief overview of the animal trials is provided in Table 2, and the detailed descriptions are given elsewhere (Nagl et al. 2012, 2014; Pestka et al. 2017; Winkler et al. 2015).

**Table 2:** Overview of animal trials. i.p.: intraperitoneal

Species	Application of DON	Timepoint of urine collection	References
Rats (♂)	single bolus (2 mg/kg b.w.) per gavage	0–24 h after application	Nagl et al. (2012)
Mice (♂)	single i.p. injection (1 mg/kg b.w.)	2 h after application	Pestka et al. (2017)
Pigs (♂)	single bolus (74 µg/kg b.w.) per gavage	0–24 h after application	Nagl et al. (2014)
Cows (♀)	via feed (5.2 mg DON/kg dry mass) for 13 weeks	At the end of the trial	Winkler et al. (2015)

## Results and discussion

### Chronology and preliminary analysis of animal urine

**Fig. 3:**
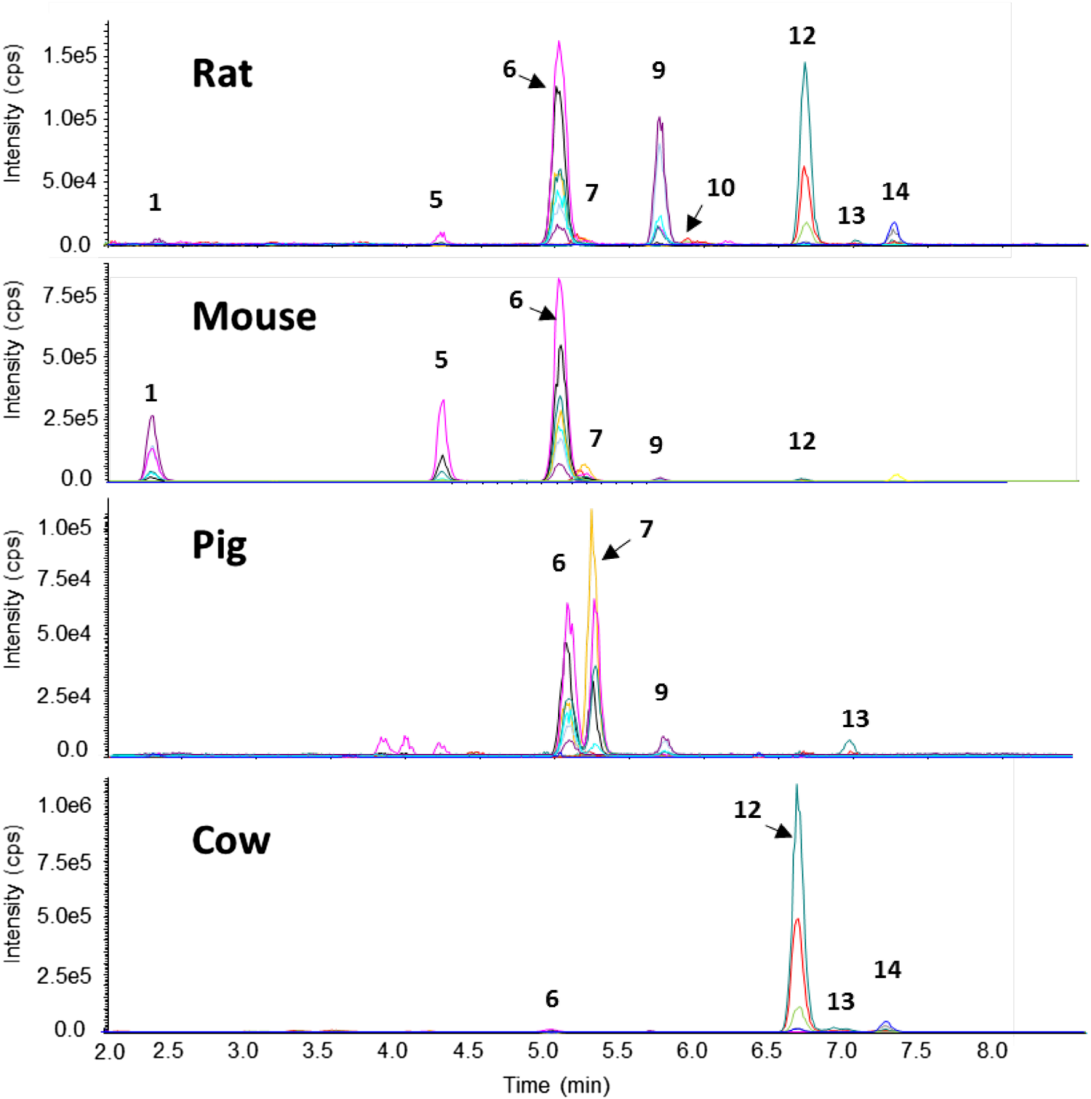
LC–MS/MS chromatograms of (iso-)DON/M-glucuronides in animal urine. *1* iso-DON-8-GlcAc, *5* DON-8,15-hemiketal-8-glucuronide, *6* DON-3-GlcAc, *7* DON-15-GlcAc, *9* iso-DON-3-GlcAc, *10* unknown DOM-GlcAc b, *12* DOM-3-GlcAc, *13* DOM-15-GlcAc, *14* iso-DOM-3-GlcAc

The starting point of our work was analysis of urine samples of various previous animal trials, where DON had been administered to rats, mice, pigs, and cows, by a flat gradient LC–MS/MS method, using selected reaction monitoring (SRM) transitions (a) optimized for DON-3-GlcAc and (b) calculated for DOM glucuronides. These preliminary measurements confirmed the presence of DON-3-GlcAc in all samples and of DON-15-GlcAc in pig urine samples and hinted at the presence of DOM-3-GlcAc in urine of cows and rats. In addition, they also yielded several new peaks at the selected SRM transitions which showed different SRM intensity ratios and appeared at different (earlier and later) retention times. Rat urine contained the greatest spectrum of metabolites. Three novel DON-GlcAc (compounds 1, 5, and 9 in Fig. 3) as well as two major novel DOM-GlcAc peaks (compounds 12 and 14) and two minor novel DOM-GlcAc (compounds 10 and 13) were detected in addition to DON-3-GlcAc (compound 6) in rat urine. Detailed results are shown in Fig. 3 and Table 7 and discussed in “LC–MS/MS analysis of animal urine samples “. As isolation of several hundred microgram amounts of these compounds—which is required for structure elucidation by NMR—is not practicable, we decided to incubate DON and DOM with rat- and human liver microsomes, to isolate the formed glucuronides and compare them with the unknown glucuronides detected in animal urine samples.

### Microsome assay for glucuronidation of DON and DOM

We incubated DON and DOM with commercially available RLM or HLM and the other reagents required for glucuronidation according to Uhlig et al. (2013). A preliminary time course experiment (0.5–48 h) yielded the greatest peak areas of DON-3-GlcAc after 24 h of incubation. The compounds formed upon incubation of DON and DOM with RLM and HLM are summarized in Table 3, where the later achieved compound identification is anticipated for reasons of clarity. HPLC–MS/MS analysis of diluted aliquots of the four reaction solutions (see Figs. S1–S4 in the electronic supplementary material) indicated formation of six DON glucuronides when DON was incubated with RLM, albeit to largely different extent and at different SRM transition intensity ratios. Consistent with previously published data (Maul et al. 2012, 2015; Uhlig et al. 2013), DON-3-GlcAc was the major product and could be identified by comparison with an authentic reference standard. The second most intense product (compound 5 in Fig. S1, 5.2% of DON-3-GlcAc based on the peak area of the transition 471.1–>113.0) was at first speculated to be either DON-7-GlcAc (Maul et al. 2012, 2015) or DON-8,15-hemiketal-8-glucuronide (Uhlig et al. 2016). Eventually, comparison of retention times and SRM transition intensity ratios of compound 5 with those of a DON-8,15-hemiketal-8-glucuronide reference standard (Uhlig et al. 2013) identified compound 5 as the latter. DON-15-GlcAc, the third most intense peak (2.0% of DON-3-GlcAc), was identified based on literature reports (Nagl et al. 2014; Sarkanj et al. 2013; Uhlig et al. 2013; Warth et al. 2012) and absence of the transition 471.1–>441.1. The peak areas of the other three formed DON glucuronides (compounds 1, 2, and 9) were minute compared to DON-3-GlcAc (0.2–0.3% based on the 113.1 fragment). Interestingly, compounds 1 and 9 showed prominent 471.1–>441.1 transitions, likely arising from loss of the CH_2_O moiety attached at C-6. Conversion of DON with HLM took place to very small extent (conversion rate <5%) and only DON-15-GlcAc as major compound and DON-3-GlcAc (23% of DON-15-GlcAc) were detected, which is in line with literature data (Maul et al. 2012, 2015; Uhlig et al. 2013).

**Table 3:** Glucuronides produced by incubation of different DON metabolites with rat or human liver microsomes

Substrate	Type of microsome	Glucuronides
DON	RLM	DON-3-GlcAc (comp 6, 100%) ≫ DON-8,15-hemiketal-8-GlcAc (comp 5, 5.2%) > DON-15-GlcAc (comp 7, 2.0%) > unknown DON-GlcAc (comp 2, 0.3%) > iso-DON-3-GlcAc (comp 9, 0.2%) ~iso-DON-8-GlcAc (comp 1, 0.1%)
DON	HLM	DON-15-GlcAc (comp 7, 100%) > DON-3-GlcAc (comp 6, 23%)
DOM	RLM	DOM-3-GlcAc (comp 12, 100%) ≫ DOM-15-GlcAc (comp 13, 2.6%) > unknown DOM-GlcAc b (comp 10, 0.8%) > unknown DOM-GlcAc a (comp 4, 0.3%) > iso-DOM-3-GlcAc (comp 14, <0.2%) ~iso-DOM-8-GlcAc (comp 3, <0.2%)
DOM	HLM	DOM-15-GlcAc (comp 13, 100%) ≫ DOM-3-GlcAc (comp 12, 7.7%)
Iso-DON	RLM	iso-DON-8-GlcAc (comp 1, 100%) > iso-DON-3-GlcAc (comp 9, 13%) > iso-DON-15-GlcAc (8.0%)
Iso-DON	HLM	iso-DON-15-GlcAc (100%) > iso-DON-3-GlcAc (comp 9, 29%)
Iso-DOM	RLM	iso-DOM-8-GlcAc (comp 3, 100%) > iso-DOM-3-GlcAc (comp 14, 54%) ~iso-DOM-15-GlcAc (54%)
Iso-DOM	HLM	iso-DOM-15-GlcAc (100%) ≫ iso-DOM-3-GlcAc (comp 12, 3.1%)

In brackets: relative intensities (values given relative to the major glucuronide in each assay) based on relative peak areas of the fragment *m/z* 113.0; comp: compound

Incubation of DOM with RLM and HLM yielded analogous products to the glucuronides formed upon microsomal incubations of DON, albeit at slightly different relative intensities. The major peak (compound 12 in Fig. S3) and second most abundant product (compound 13, 2.0% of the major compound based on the transition 455.1–>113.3) formed upon conversion of DOM by RLM were tentatively identified as DOM-3-GlcAc and DOM-15-GlcAc based on SRM transitions [lack of the (M-H-30)^−^ fragment for DOM-15-GlcAc] and analogy to formation of DON-3-GlcAc and DON-15-GlcAc. Two further novel DOM-GlcAc (compounds 4 and 10) were formed to 0.3 and 0.8% relative to DOM-3-GlcAc. In addition, two very minor DOM-GlcAc peaks (compounds 3 and 14) were detected only at the transitions 455.1–>425.1 and 455.1–>249.1).

Similar to incubation of DON with HLM, DOM-15-GlcAc was the major glucuronide formed upon incubation of DOM with HLM, followed by DOM-3-GlcAc (7.7% of DOM-15-GlcAc). However, the glucuronidation rate of DOM with HLM was much higher than that of DON (see Figs. S2 and S4). The relatively fast formation of DOM glucuronides upon incubation of the far less toxic DOM with HLM indicated that the used preparation of HLM was in principle active, but unsuited for quantitative glucuronidation of DON. Low activity of HLM for glucuronidation of DON, but good activity for glucuronidation of 4-tri-fluoromethylumbelliferone as a reference substance had already been reported by Maul et al. (2012). In addition, greater glucuronidation of DOM compared to DON had been observed in pigs, where DON glucuronides made up approximately 40% of total urinary DON, whereas enzymatic hydrolysis increased the urinary DOM concentrations by a factor of 3.5 on average (Nagl et al. 2014).

### Semi-preparative isolation of glucuronides produced in the microsome assay

In line with the HPLC–MS/MS chromatograms of the diluted incubation solutions of DON and DOM incubated with RLM and HLM, six DON-GlcAcs were collected when DON was incubated with RLM and six DOM-GlcAcs were obtained from the reaction mixture of DOM with RLM. As fractions of equal volume were collected in short intervals, compounds eluted in more than one fraction. For most compounds, both pure and mixed fractions were collected. To give an overview of the obtained glucuronides, a chromatogram containing all glucuronides isolated in the course of the first microsome assays for production of DON glucuronides (compounds 1, 2, 5, 6, 7, and 9) and DOM glucuronides (compounds 3, 4, 10, 12, 13, and 14) is shown in Fig. 2. Solutions of the individual glucuronides were pooled in such a way as to obtain similar signal intensities for all compounds. In Table 4, the collection intervals of the fractions containing pure compounds and, if pure fractions could not be collected for a compound, of the fractions of the highest purity are given. For reasons of clarity, the compound identification which was later achieved by NMR measurements is mentioned already at that stage. Interestingly, in addition to the parent compounds DON and DOM, minute amounts of one later eluting compound displayed at the same SRM transitions, but at different intensity ratios was collected for each substance (compounds 11 and 16).

**Table 4:** Semi-preparative HPLC isolation of glucuronides and parent compounds from microsome assay solutions. ref.: reference

Compound number^a^	Compound name (method of identification)	Production	Fractions^b^	Collection time (min)
1	Iso-DON-8-GlcAc (NMR)	DON RLM	6	3.5–3.7
2	Unknown DON-GlcAc	DON RLM	9–11	4.1–4.7
5	DON-8.15-hemiketal-8-GlcAc (ref. standard)	DON RLM	15, 16	5.3–5.7
6	DON-3-GlcAc (ref. standard)	DON RLM	18, 19	5.9–6.3
9	Iso-DON-3-GlcAc^c^ (NMR)	DON RLM	25	7.3–7.5
7	DON-15-GlcAc^d^ (literature)	DON HLM	20	6.3–6.5
8	DON (ref. standard)	DON RLM, DON HLM	22	6.7–6.9
11	Iso-DON^e^ (NMR)	DON RLM, DON HLM	26	7.5–7.7
3	Iso-DOM-8-GlcAc^f^ (NMR)	DOM RLM	8	3.9–4.1
4	Unknown DOM-GlcAc a	DOM RLM	11	4.5–4.7
10	Unknown DOM-GlcAc b	DOM RLM	23	6.9–7.1
12	DOM-3-GlcAc (NMR)	DOM RLM	27, 28	7.7–8.1
13	DOM-15-GlcAc (NMR)	DOM HLM	30	8.3–8.5
14	Iso-DOM-3-GlcAc^g^ (NMR)	DOM RLM	36	9.5–9.7
15	DOM (ref. standard)	DOM RLM, DOM HLM	34	9.1–9.3
16	Iso-DOM^h^ (NMR)	DOM RLM, DOM HLM	37	9.7–9.9

^a^Number of the compound in Fig. 2

^b^Fractions containing pure compound unless stated otherwise

^c−h^Only collected in mixed fractions. Impurities: ^c^ DON and iso-DON, ^d^ DON-3-GlcAc, ^e^ iso-DON-3-GlcAc (DON RLM), DON (DON HLM), ^f^ unknown DOM-GlcAc a, ^g^ DOM, ^h^ DOM

To assess the relevance of the six glucuronides formed for DON and DOM, respectively, we compared the retention times and SRM transition intensity ratios of the isolated compounds with the glucuronides detected in animal urine samples (see Figs. 2, 3). In addition to DON-3-GlcAc that was detected in all samples and DON-15-GlcAc in pig urine, DON-8,15-hemiketal-8-GlcAc and compounds 1, 9, 12, 13, and 14 occurred in animal urine samples. As the amounts of glucuronides obtained in the first assays were not sufficient for structure elucidation by NMR, our next step was to verify if DON and DOM were the aglycones of the isolated glucuronides.

### Enzymatic hydrolysis

Each isolated glucuronide was subjected to enzymatic hydrolysis with β-glucuronidase. Surprisingly, two DON-derived glucuronides (compounds 1 and 9) and two DOM-based glucuronides (compounds 3 and 14) did not yield DON or DOM, but later eluting compounds appearing at the same SRM transitions, but at different intensity ratios. These substances were identical to the compounds 11 and 16 isolated in the course of the first microsome assays. Literature research indicated that the DON isomer might be iso-DON (3,8,15-trihydroxy-12,13-epoxytrichothec-8-en-7-one, see Fig. 1), a compound originally identified as side product of baking bread with DON-contaminated flour (Greenhalgh et al. 1984). Complete hydrolysis was obtained for DON-3-GlcAc, DON-15-GlcAc, DOM-3-GlcAc, and DOM-15-GlcAc and for the most likely analogous compounds 9 and 14. The other analogous compounds 1 and 3 were partially hydrolyzed (<40% cleavage). Partial hydrolysis was also observed for compound 2 (ca. 70%), compounds 4 and 10 (<30% cleavage), and DON-8,15-hemiketal-8-GlcAc (<10%). The resistance of DON-8,15-hemiketal-8-GlcAc to enzymatic hydrolysis had already been suggested by Uhlig et al. (2013).

The finding that some glucuronides present in animal urine (iso-DON-based compound 1 and DON-8,15-hemiketal-8-GlcAc) are at best partially cleaved upon enzymatic hydrolysis highlights the importance of proper biomarker method development. Most likely, compound 1 and DON-8,15-hemiketal-8-GlcAc have escaped detection whenever enzymatic hydrolysis had been employed. Likewise, iso-DON- and iso-DOM- glucuronides have never been quantified in hydrolysis methods unless iso-DON and iso-DOM accidentally co-eluted in the employed LC–MS/MS method. Still, in the case of co-elution, quantification was falsified by different ionization and fragmentation intensities of the isomeric compounds.

### Production and purification of iso-DON and iso-DOM

Occurrence of compounds 1 and 9 in urine of mice, rats, and/or pigs, and presence of compound 14 in rat- and cow urine underlined the relevance of these assumed iso-DON- and iso-DOM glucuronides. Therefore, the next step was to produce iso-DON and iso-DOM to (a) confirm the structure of the unconjugated DON- and DOM-metabolites and (b) generate the substrates for microsome assays which were required to elucidate the structures of the iso-DON- and iso-DOM glucuronides.

First, we attempted to produce iso-DON and iso-DOM by heating solid DON and DOM, respectively, at 160 °C for 1 and 2 h as described by Greenhalgh et al. (1984). However, after heating for 1 h, formation of iso-DON and iso-DOM was <1% based on UV-detection at 220 nm. Several side products were produced, too (Bretz et al. 2006). Heating for 2 h slightly increased the proportion of formed iso-DON and iso-DOM to <2%, but also increased the number and concentration of side products. An alternative way of producing iso-DON is by use of sodium methoxide, a chemical often used in organic chemistry for achieving isomerization of compounds. Here, the conversion rate of DON to iso-DON was approximately 8% in the used protocol. Prolonged incubation or increase in sodium methoxide concentration did not enhance iso-DON formation, but increased the number and concentration of undesired side products (data not shown). Still, formation of at least two unidentified side products that were displayed at the SRM transition 355.1–>59.0 and eluted slightly before and after iso-DON in preparative chromatography could not be avoided. Therefore, one pure iso-DON fraction and two impure fractions containing iso-DON and one of each side products were obtained upon semi-preparative chromatography. Analytical HPLC separation and collection of the iso-DON peak were required to isolate iso-DON from these mixed fractions. To obtain at least 5 mg of iso-DON, the amount of DON regained upon preparative chromatography of the reaction mixture was again subjected to sodium methoxide treatment. In sum, three cycles of iso-DON production were carried out and 6.2 mg of iso-DON was produced.

Iso-DOM formation upon incubation of DOM with sodium methoxide was <2% based on UV detection at 220 nm. However, under the conditions employed for conversion of DON to DOM by means of the anaerobic bacterium BBSH 797 (Schwartz-Zimmermann et al. 2014), one side product was formed which had the same retention time and SRM transition intensity ratios as the aglycone of compounds 3 and 14. Hence, we purified this compound from a mixed DOM/iso-DOM fraction collected upon preparative isolation of DOM according to Schwartz-Zimmermann et al. (2015). In sum, 4.5 mg of iso-DOM were obtained.

### Production and purification of DOM-, iso-DON-, and iso-DOM glucuronides

To enable structure elucidation or confirmation by NMR, compounds 1, 3, 9, 12, 13, and 14 had to be produced at larger scale. As already known from the first microsome assays, compounds 12 and 13 (supposed to be DOM-3-GlcAc and DOM-15-GlcAc) could be produced by incubation of DOM with RLM and HLM, respectively. As expected, conversion of DOM with RLM and semi-preparative isolation yielded majorly DOM-3-GlcAc (2.1 mg), but also a mixed fraction containing DOM-3-GlcAc and DOM-15-GlcAc. Pure DOM-15-GlcAc was obtained as major metabolite when DOM was incubated with HLM. Again, a mixed fraction containing DOM-3-GlcAc and DOM-15-GlcAc was collected, and both mixed fractions were purified again. Finally, 1.3 mg of DOM-15-GlcAc was obtained.

For production of the iso-DON based compounds 1 and 9 and of the iso-DOM-derived glucuronides 3 and 14, iso-DON and iso-DOM were incubated with RLM. The results are summarized in Table 3. Interestingly, in both cases, a third glucuronide was formed in addition to the expected compounds. These novel glucuronides eluted in front of compound 9 in the iso-DON assay and in front of compound 14 in the iso-DOM reaction batch. LC–MS/MS analysis and LC–HR-MS/MS spectra (see below and supplementary material) clearly showed the absence of the fragment *m/z* 441.1 for the novel iso-DON-GlcAc and the absence of the fragment *m/z* 425.1 for the novel iso-DOM-GlcAc which, in both cases, suggested conjugation at C-6. Judging from the transition 471.1–>113.1 in HPLC–MS/MS analysis, compound 1 was the major product of incubating iso-DON with RLM, followed by compound 9 (13%) and the suspected iso-DON-15-GlcAc (8%). Conversion of iso-DOM with RLM yielded compound 3 as major product and similar formation of compound 14 and the assumed iso-DOM-15-GlcAc (both 54% based on the transition *m/z* 455.1–>113.1). To confirm the proposed structures of the iso-DON/M-15-GlcAc, iso-DON and iso-DOM were incubated with HLM in small scale. As observed for DON and DOM incubated with HLM, the assumed 15-glucuronides were the major products, followed by compound 9 (29% based on the transition *m/z* 471.1–>113.1) in the case of iso-DON and compound 14 (3% based on the transition *m/z* 455.1–>113.1) in the case of iso-DOM. Similarly, the conversion rate with HLM was much greater for iso-DOM (ca. 70%) than for iso-DON (ca. 10%). Considering the analogy to glucuronidation of DON and DOM by HLM which yielded DON/DOM-15- and -3-glucuronides, formation of the later eluting iso-DON- and iso-DOM glucuronides (compounds 9 and 14), but not of the earlier eluting compounds 1 and 3 strongly suggested compounds 9 and 14 to be the iso-DON/iso-DOM-3-glucuronides and compounds 1 and 3 to be the iso-DON/iso-DOM-8-glucuronides. For unequivocal identification by NMR spectroscopy, compound 1 which partly co-eluted with reagent compounds and compound 9 which eluted closely to iso-DON had to be isolated from their mixed fractions by analytical HPLC. In sum, 2.1 mg of compound 1, 0.5 mg of iso-DON-15-GlcAc, and 0.76 mg of compound 9 were obtained. Semi-preparative chromatography of the incubation mixture of iso-DOM with RLM yielded 2.8 mg of compound 1, 0.7 mg of iso-DOM-15-GlcAc, and 1.0 mg of compound 14.

### NMR

Compounds 1, 3, 9, 11, 12, 13, 14, and 16 were analyzed by 1D (^1^H and ^13^C) and 2D (H,H-COSY, H,C-HSQC, and H,C-HMBC) NMR measurements, and complete assignments for all signals were achieved. Tables 5 and 6 give the ^1^H and ^13^C chemical shifts and multiplicities. All NMR spectra are given in the supplementary material. In Table 4, the compound names are assigned to the compound numbers; Fig. 1 shows the numbering of the skeletons.

**Table 5:** ^1^H NMR chemical shifts (ppm) and multiplicities (J, Hz); spectra were recorded in methanol-d_4_

	Iso-DON	Iso-DON-3-GlcAc	Iso-DON-8-GlcAc	Iso-DOM	Iso-DOM-3-GlcAc	Iso-DOM-8-GlcAc	DOM-3-GlcAc	DOM-15-GlcAc
H-2	3.34 (d)	3.59 (d, 4.5)	3.35 (d, 4.4)	3.96 (d, 4.2)	4.20 (d, 4.4)	3.99 (d, 4.2)	4.34 (m)	4.14 (m)
H-3	4.30 (dt, 11.1, 4.5)	4.58 (dt, 10.8, 4.4)	4.31 (dt, 11.0, 4.4)	4.06 (dt, 11.1, 4.5)	4.36 (dt, 11.0, 4.6)	4.07 (dt, 11.0, 4.5)	4.34 (m)	4.14 (m)
H-4	2.49 (dd, 14.6, 4.5) 1.92 (dd, 14.6, 11.1)	2.61 (dd, 14.6, 4.5) 1.94 (dd, 14.7, 10.9)	2.34 (dd, 14.6, 4.4) 1.93 (dd, 14.6, 11.1)	2.51 (dd, 14.5, 4.7) 1.80 (dd, 14.5, 11.2)	2.63 (dd, 14.5, 4.7) 1.81 (dd, 14.4, 11.1)	2.31 (dd, 14.4, 4.7) 1.83 (dd, 14.4, 11.2)	2.59 (m) 1.85 (m)	2.43 (m) 1.84 (m)
H-7	–	–	–	–	–	–	4.61 (s)	4.65 (s)
H-10	2.98 (m) 2.43 (d, 18.9)	2.97 (m) 2.42 (d, 18.9)	3.12 (dd, 19.3, 3.5) 2.53 (dd, 19.2, 1.6)	2.94 (m) 2.36 (d, 18.8)	2.93 (m) 2.36 (d, 19.0)	3.15 (d, 18.9) 2.47 (dd, 19.0, 1.8)	6.59 (dq, 5.9, 1.5)	6.58 (dq, 5.9, 1.5)
H-11	4.81 (m)	4.79 (m)	4.76 (m)	4.86 (dd, 3.8, 2.4)	4.85 (m)	4.77 (m)	4.90 (m)	4.97 (d, 5.9)
H-13	2.80 (d, 4.4) 2.77 (d, 4.4)	2.80 (d, 4.5) 2.76 (d, 4.5)	2.83 (d, 4.3) 2.81 (d, 4.3)	4.91 (d) 4.70 (d, 0.9)	4.94 (s) 4.71 (s)	4.96 (s) 4.71 (s)	5.18 (s) 5.01 (s)	5.17 (s) 5.01 (s)
H-14 (5-CH_3_)	1.30 (s)	1.30 (s)	1.26 (s)	1.61 (s)	1.62 (s)	1.56 (s)	1.42 (s)	1.41 (s)
H-15 (CH_2_OH)	3.77 (d, 11.9) 3.74 (d, 11.9)	3.77 (s)	3.84 (d, 11.7) 3.78 (d, 11.7)	3.77 (d, 11.8) 3.73 (d, 11.8)	3.78 (d, 12.0) 3.77 (d, 12.0)	3.93 (d, 11.8) 3.79 (d, 11.8)	3.75 (s)	4.28 (d, 10.3) 3.61 (d, 10.3)
H-16 (9-CH_3_)	1.85 (m)	1.84 (m)	2.03 (m)	1.83 (m)	1.82 (m)	2.05 (s)	1.81 (m)	1.81 (m)
1′	–	4.40 (d, 7.9)	4.55 (m)	–	4.39 (d, 7.8)	4.38 (d, 7.4)	4.44 (d, 7.8)	4.04 (d, 7.7)
2′-5′	–	3.57–3.27 (m)	3.61–3.41 (m)	–	3.56–3.28 (m)	3.50–3.39 (m)	3.70–3.31 (m)	3.52–3.07 (m)

**Table 6:** ^13^C NMR chemical shifts (ppm); spectra were recorded in methanol-d_4_

	Iso-DON	Iso-DON-3-GlcAc	Iso-DON-8-GlcAc	Iso-DOM	Iso-DOM-3-GlcAc	Iso-DOM-8-GlcAc	DOM-3-GlcAc	DOM-15-GlcAc
C-2	82.3	82.1	82.4	82.6	82.3	82.5	82.5	82.8
C-3	69.9	75.9	69.8	70.5	76.0	70.4	76.7	70.4
C-4	45.1	42.2	45.2	45.8	42.9	45.8	42.8	45.3
C-5	46.9	46.7	47.1	49.1	48.7	49.3	49.7	50.1
C-6	57.7	57.8	59.0	59.3	59.5	60.8	54.3	54.2
C-7	196.8	196.8	198.4	196.6	196.6	200.2	75.9	75.6
C-8	144.8	144.8	146.7	144.8	144.9	148.1	202.4	203.6
C-9	125.9	126.1	146.3	125.2	125.4	147.8	137.1	137.0
C-10	34.6	34.8	36.0	34.6	34.7	36.3	139.9	140.5
C-11	72.5	72.8	72.5	73.1	73.3	72.9	71.9	72.1
C-12	67.6	67.3	67.5	155.3	154.7	155.2	154.6	155.0
C-13	50.3	50.2	50.6	107.5	107.7	107.7	108.0	108.1
C-14 (5-CH_3_)	14.7	14.8	14.9	20.1	20.2	20.3	20.4	20.4
C-15 (CH_2_OH)	64.4	64.4	64.4	64.5	64.5	64.8	62.0	70.1
C-16 (9-CH_3_)	17.1	17.1	18.9	17.1	17.1	19.1	15.6	15.7
1′	–	103.5	105.1	–	103.2	105.9	103.6	105.0
2′	–	75.0	75.5	–	75.0	75.3	74.9	75.2
3′	–	78.1	77.8	–	78.0	78.1	77.8	77.7
4′	–	73.8	73.3	–	73.8	73.2	73.5	73.6
5′	–	76.6	77.1	–	76.4	76.7	76.6	76.4
6′ (COOH)	–	177.1	174.5	–	177.1	176.6	174.8	176.9

Compound 11 was identified as iso-DON by comparison of the ^1^H and ^13^C NMR data with those given by Greenhalgh et al. (1984); analysis of its 2D spectra confirmed the assignments given there, except for the erroneous shifts for C-8 and C-9 (144.8 and 125.2 ppm, resp.). For compound 16, the spectral features of the A ring (C-6 to C-11; identified by their long-range C–H correlations) as well as C-15 and C-16 were found very similar to iso-DON. Another HMBC relation from C-6 (59.3 ppm) identified the CH_3_ group in position 14, the protons of which also show long-range correlations to 155.3 (C-12) and 45.8 ppm (C-4). Starting from the latter position, the remains of the C ring (C-2 to C-4) were identified mainly by means of the COSY, which connects H-4a, H-4b, H-3, and H-2 to a pattern very characteristic for DON-derived trichothecenes. HMBC correlations from H-2 (3.96 ppm) to 107.5 (C-13) and 155.3 ppm (C-12) define the deepoxy substructure, and another one to 73.1 ppm (C-11) finally shows the intact B ring. Thus, compound 16 has been positively identified as iso-DOM.

In all analyzed glucuronides, the attachment point of the glucuronic acid unit to the respective parent structure (iso-DON, DOM, or iso-DOM) could unambiguously be proven by HMBC long-range correlation of the carbohydrate’s H-1′ peak (anomeric proton) to the signal of the trichothecene carbon carrying the glucuronic acid moiety: C-8 (146.7 ppm for compound 1 and 148.1 ppm for compound 3); C-3 (75.9 ppm for compound 9, 76.7 ppm for compound 12, and 76.0 ppm for compound 14); or C-15 (70.1 ppm for compound 13). In addition, a characteristic downfield shift of 6-8 ppm was observed, where the attachment carbon is aliphatic (C-3 or C-15); in case of the C-8 glucuronides of iso-DON and iso-DOM, the most prominent effect is a 20 ppm downfield shift of the neighbouring C-9 signal due to the severely reduced electron-donating effect of the C-8-attached oxygen atom. Apart from these differences, the spectral features of all glucuronides closely resemble those of their parent compounds 11, 15, or 16.

### HPLC–HR-MS and HPLC–HR-MS/MS analysis

The exact mass of all isolated DON- and iso-DON glucuronides is 472.1581 Da, the exact mass of all obtained DOM- and iso-DOM glucuronides 456.1632 Da. For all 16 glucuronides, the determined accurate mass deviated by less than 6 ppm from the exact mass. HR-MS/MS spectra of all isolated compounds are given in the electronic supplementary material. For most compounds, a CE of −30 eV was best suited in terms of relative intensity of precursor- and product ions. However, DON-3-GlcAc and DON-15-GlcAc required a CE of −35 eV for proper fragmentation of the precursor ions. Contrary to that, the iso-DON- and iso-DOM-3- and -8-glucuronides fragmented very easily, resulting in complete disappearance of the deprotonated ion at −30 eV. Hence, for these compounds, MS/MS spectra at −25 eV are shown. In general, the intensity of the fragments is highly dependent on the CE. Hence, we focused on the presence or absence of fragments rather than on similar fragment intensity patterns when we compared our mass spectra with literature spectra.

Fragmentation spectra of DON-3-GlcAc were similar to those published in the literature (Maul et al. 2012; Sarkanj et al. 2013; Uhlig et al. 2013; Warth et al. 2012). In all spectra, the fragment *m/z* 441.1 was detected which is indicative of the presence of, e.g., an unconjugated –CH_2_OH group in the molecule which can be lost as CH_2_O in the collision cell. In addition, the DON-specific fragments *m/z* 265.1, 247.1, and 229.1 as well as the glucuronide-based fragments *m/z* 175.1 and 113.0 were visible in all spectra of DON-3-GlcAc—provided that these ions were included in the selected scan range. Fragments formed by neutral loss of 30 Da, corresponding to loss of CH_2_O including C-15 (*m/z* 441.1 and 425.1), were very prominent in the fragmentation spectra of the 3- and 8 glucuronides of iso-DON and iso-DOM. Likewise, the fragment ions of *m/z* 265.1 (iso-DON-GlcAc) and 249.1 (iso-DOM-GlcAc), resulting from neutral loss of the glucuronide moiety and of formaldehyde, were among the dominating fragments. Easily induced fragmentation compared to DON-3-GlcAc and DON-15-GlcAc (indicated by low intensity of the precursor ion in the product ion spectrum) and prominent fragment ions of *m/z* 441.1 and 265.1 were also reported for the previously published tentatively identified DON-7-GlcAc (Maul et al. 2012; Sarkanj et al. 2013). In line with the published spectra, the assumed DON-7-GlcAc showed intense fragments of *m/z* 441.1, 265.1, and 229.1 and other prominent fragments of *m/z* 247.1 and 217.1. In addition, putative DON-7-GlcAc eluted after DON-15-GlcAc in reversed phase chromatography. Hence, considering the elution order in reversed phase chromatography and the mass spectra, it is likely that the previously tentatively identified DON-7-GlcAc is actually iso-DON-3-GlcAc.

Neutral loss of 30 Da was also observed in the spectrum of DOM-3-GlcAc, but the fragment *m/z* 425.1 had very low intensity. The major fragments generated for DOM-3-GlcAc were the glucuronide-derived ions *m/z* 113.0 and 193.1, followed by 131.0. Consistent with literature spectra, fragmentation spectra of DON-15-GlcAc, DOM-15-GlcAc, iso-DON-15-GlcAc, and iso-DOM-15-GlcAc lacked the fragment *m/z* 441.1, which confirmed conjugation of these compounds at C-15. Consistent with published mass spectra of DON-15-GlcAc (Sarkanj et al. 2013; Uhlig et al. 2013), the major fragment ions of DON-15-GlcAc were *m/z* 265.1, 193.1, 150.0, and 113.0. The glucuronide-derived fragments *m/z* 193.1 and 113.0 were also the major ions in the mass spectra of DOM-15-GlcAc, iso-DON-15-GlcAc, and iso-DOM-15-GlcAc.

Mass spectra recorded for DON-8,15-hemiketal-8-GlcAc were similar to the spectra provided in the literature (Sarkanj et al. 2013; Uhlig et al. 2013). Due to the 8,15-hemiketal structure, they completely lacked the *m/z* 441.1 fragment. At a CE of −30 eV, the precursor ion 471.1 and the glucuronide-based fragment 113.0 were the major ions, followed by the fragment ions 175.0, 265.1, 247.1, and 129.0. The unidentified DOM-GlcAc b (compound 10) fragmented similar to DON-8,15-hemiketal-8-GlcAc. At −30 eV, the precursor ion *m/z* 455.1 and the fragment ion *m/z* 113.0 showed the highest intensity, followed by the ions *m/z* 249.1, 231.1 (analogous to 265.1 and 247.1), and 129.0. As also the chromatographic elution order was analogous for DON-8,15-hemiketal-8-GlcAc and compound 10, we tentatively assigned the structure DOM-8,15-hemiketal-8-GlcAc to compound 10.

### HPLC–MS/MS analysis

Optimization of SRM parameters showed the transitions 471.1- > 193.1 and 455.1–>193.1 to be of the highest intensity for all four (iso-)DON/M-15-glucuronides. In addition, testing of various CEs revealed that the transitions 471.1–>441.1 and 455.1–>425.1 yielded the highest signals for iso-DON/M-3- and 8-glucuronides at a relatively low CE of −25 eV. The optimized SRM parameters are summarized in Table 1.

### Animal trials

Representative chromatograms of urine samples collected from rats, mice, and pigs treated with one single bolus of DON and from cows fed with DON-contaminated feed for 13 weeks are displayed in Fig. 3. For reasons of clarity of chromatograms, the noisier DON- and DOM-SRM transitions are not shown. The four analyzed mouse urine samples had a highly similar DON metabolite pattern. Similarly, low variation in the DON metabolite pattern was observed for the four cow urine samples. For the four rat urine samples, the intensity ratios of the main metabolites were slightly variable. The glucuronide pattern in the four investigated pig urine samples showed some variability in the relative peak areas of the individual glucuronides.

Exact quantification of glucuronides in biological samples requires a proper validation of the LC–MS/MS method which was out of the scope of this article. At this stage, peak areas of the most intense SRM transitions for each analyte were compared to obtain an overview of the glucuronidation pattern (see Table 7). In all of the measured rat urine samples, DON-3-GlcAc was the dominant glucuronide. DOM-3-GlcAc and iso-DON-3-GlcAc ranked second and third, showing similar, but slightly variable intensities. The fourth significant glucuronide was iso-DOM-3-GlcAc. Small peaks of DON-8,15-hemiketal-8-GlcAc, iso-DON-8-GlcAc, DON-15-GlcAc, DOM-15-GlcAc and of the unknown DOM-GlcAc b (tentatively identified as DOM-8,15-hemiketal-8-GlcAc) were detected in some of the investigated samples. In addition, minor concentrations of unconjugated iso-DON were present. In all mouse urine samples, DON-3-GlcAc was the major glucuronide, followed by DON-8,15-hemiketal-8-GlcAc and iso-DON-8-GlcAc. DON-15-GlcAc and iso-DON-3-GlcAc occurred in traces. Only traces of DOM-3-GlcAc were detected which is likely result using i.p. administration rather than oral gavage. All pig urine samples contained DON-15-GlcAc as main and DON-3-GlcAc as second most important glucuronide. Iso-DON-3-GlcAc occurred in three of the four analyzed urine samples. However, as previously published for DON-15-GlcAc and DON-3-GlcAc (Nagl et al. 2014), there were inter-individual differences in the relative peak intensities of the three glucuronides. Small peaks of DOM-15-GlcAc could be detected in two samples. The high microbial activity in the ruminant gastro-intestinal system leads to exhaustive conversion of DON to DOM (Winkler et al. 2015). Consequently, the dominant metabolite in cow urine was DOM-3-GlcAc, followed by iso-DOM-3-GlcAc. DON-3-GlcAc, DOM-15-GlcAc, and iso-DON-3-GlcAc were minor metabolites.

**Table 7:** Occurrence of DON and its metabolites in animal urine

Analyte	Rat	Mouse	Pig	Cow
(1) Iso-DON-8-GlcAc	(+)	++		
(5) DON-8,15-hemiketal-8-GlcAc	+	++		
(6) DON-3-GlcAc	+++	+++	+++	+
(7) DON-15-GlcAc	(+)	(+)	+++	
(8) DON	+++	++++	++++	
(9) Iso-DON-3-GlcAc	++	(+)	+	+
(10) Unknown DOM-GlcAc b	(+)			
(11) Iso-DON	+			
(12) DOM-3-GlcAc	+++	(+)		++++
(13) DOM-15-GlcAc	(+)		+	+
(14) Iso-DOM-3-GlcAc	+			++
(15) DOM	+			(+)

The number in brackets corresponds to the number of the compound in Fig. 2

(*+*) trace levels, *+* minor levels, *++* moderate levels, *+++* high levels, *++++* dominant compound

The extent of in vivo glucuronidation corresponded well with the reported relative glucuronidation activities in microsome assays (Maul et al. 2012, 2015). The glucuronidation rate was greatest in cows, where only traces of DOM were detected. In rat urine, peak areas of the major glucuronides were similar or slightly greater than peak areas of DON. In line with greater glucuronidation of DOM compared to DON (Nagl et al. 2014), peak areas of DOM-3-GlcAc were 3–4 times greater than those of DOM. In pig and mouse urine, the major part of DON remained unconjugated. As already discussed by Maul et al. (2012) on the basis of in vitro data, the ready glucuronidation in cows and rats might contribute to the lower sensitivity of these species to DON compared to pigs, even if the main reasons are most likely a high degree of deepoxidation (cows and rats) and a relatively low bioavailability of DON (rats). To gain further insight into the reasons for species specific differences in DON sensitivity, toxicity assessment of iso-DON and iso-DOM is warranted.

To sum up, the novel DON- and DOM-derived glucuronides DOM-3-GlcAc, iso-DON-3-GlcAc, and iso-DON-8-GlcAc are major metabolites in animal urine. Iso-DON-3-GlcAc, most likely previously tentatively identified as DON-7-GlcAc, is an important metabolite in urine of rats and occurred in traces in urine of mice, pigs, and cows. In addition, DON-8,15-hemiketal-8-GlcAc could be detected in animal urine for the first time.

## Conclusion

Prior to this work, the only DON glucuronides identified beyond doubt in human and animal urine samples had been DON-3-GlcAc and DON-15-GlcAc. Analysis of urine samples collected from DON-treated rats, mice, pigs, and cows by a generic LC–MS/MS method revealed the presence of seven additional DON- and DOM glucuronides, of which four seemed to be major DON metabolites in at least one of the investigated animal species. By incubating DON, DOM and their newly produced isomers iso-DON and iso-DOM with rat and human liver microsomes, by performing semi-preparative isolation, HPLC–HR-MS/MS, and NMR characterization of the reaction products, and by comparing with one previously characterized reference standard, we eventually identified six of the novel DON- and DOM-based glucuronides in animal urine. One major novel compound detected in rat, mouse, and pig urine was iso-DON-3-GlcAc, which had most likely previously been misidentified as DON-7-GlcAc.

The presence of iso-DON glucuronides as important DON metabolites in urine of mice and rats, the detection of iso-DOM glucuronides in urine of rats and cows and the occurrence of DON-8,15-hemiketal-8-GlcAc in urine of mice have implications for DON-biomarker analysis methods. All of these glucuronides escape detection in the conventional methods based on enzymatic hydrolysis and detection of released DON and DOM. Inclusion of iso-DON and iso-DOM in the analytical method would solve the problem for iso-DON-3-GlcAc and iso-DOM-3-GlcAc which are quantitatively hydrolyzed. Detection of iso-DON-8-GlcAc and DON-8,15-hemiketal-8-GlcAc, important DON metabolites in mouse urine that are at best partially hydrolyzed with β-glucuronidase, requires inclusion of the glucuronide SRM transitions into the LC–MS/MS method. To conclude, by discovering, producing, and structurally elucidating several novel DON- and DOM glucuronides, we enhanced the current knowledge on DON metabolism by different animal species and paved the way for analyzing these compounds in animal urine. Future quantitative analysis of the novel glucuronides in animal urine will show their biological relevance. In addition, studies on the toxicity of iso-DON and its derivatives are warranted.

## Electronic supplementary material

Below is the link to the electronic supplementary material.

Supplementary material 1 (DOCX 2039 kb)

## Abbreviations

CE: Collision energy
DOM: Deepoxy-deoxynivalenol
DON: Deoxynivalenol
GlcAc: Glucuronide
HLM: Human liver microsomes
HPLC–HR-MS/MS: High-performance liquid chromatography–high-resolution tandem mass spectrometry
HPLC–MS/MS: High-performance liquid chromatography–tandem mass spectrometry
I.p.: Intraperitoneal
Iso-DOM: Iso-deepoxy-deoxynivalenol
Iso-DON: Iso-deoxynivalenol
*m/z*: Mass-to-charge ratio
NMR: Nuclear magnetic resonance spectroscopy
RLM: Rat liver microsomes
SRM: Selected reaction monitoring
