# Supplementary material for: Glucuronidation of deoxynivalenol (DON) by different animal species: identification of iso-DON glucuronides and iso-deepoxy-DON glucuronides as novel DON metabolites in pigs, rats, mice, and cows
Source: Arch Toxicol. 2017 Jun 21;91(12):3857–72. doi: 10.1007/s00204-017-2012-z (PMC5719127; doi:10.1007/s00204-017-2012-z)

**Glucuronidation of deoxynivalenol (DON) by different animal species:** **Identification of** **iso-DON glucuronides and iso-deepoxy-DON glucuronides as novel DON metabolites in pigs, rats, mice and cows**

**Archives of Toxicology**

Heidi E. Schwartz-Zimmermann^*^, Christian Hametner, Veronika Nagl, Iris Fiby, Lukas Macheiner, Janine Winkler, Sven Dänicke, Erica Clark, James J. Pestka, Franz Berthiller

* Correspondence to: Heidi E. Schwartz-Zimmermann, Christian Doppler Laboratory for Mycotoxin Metabolism and Center for Analytical Chemistry, Department of Agrobiotechnology (IFA-Tulln), University of Natural Resources and Life Sciences, Vienna (BOKU), Konrad-Lorenz-Str. 20, 3430 Tulln, Austria. E-mail address: [heidi.schwartz@boku.ac.at](mailto:heidi.schwartz@boku.ac.at)

**HPLC-MS/MS chromatograms of microsome assay solutions:**

**
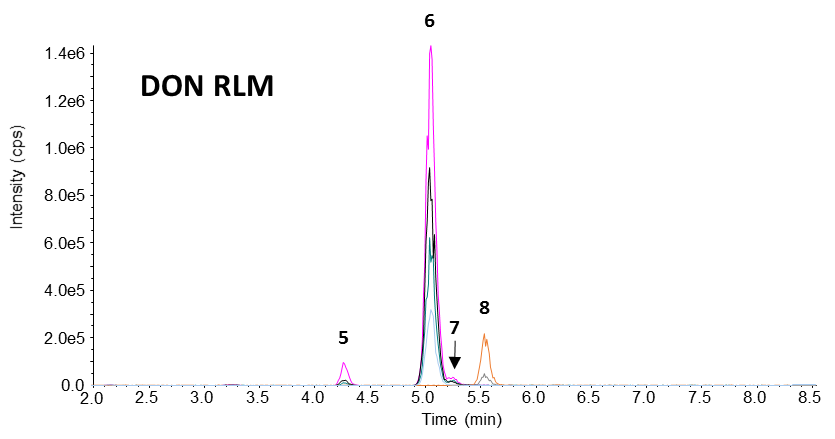
**

Figure S1: LC-MS/MS chromatogram of DON and its glucuronides isolated after incubation of DON with RLM. 5: DON-8,15-hemiketal-8-GlcAc, 6: DON-3-GlcAc, 7: DON-15-GlcAc, 8: DON.

**
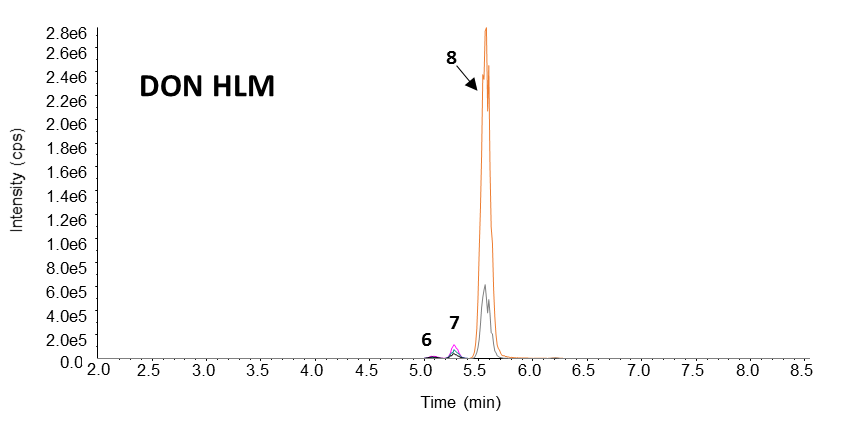
**

Figure S2: LC-MS/MS chromatogram of DON and its glucuronides isolated after incubation of DON with HLM. 6: DON-3-GlcAc, 7: DON-15-GlcAc, 8: DON.

**
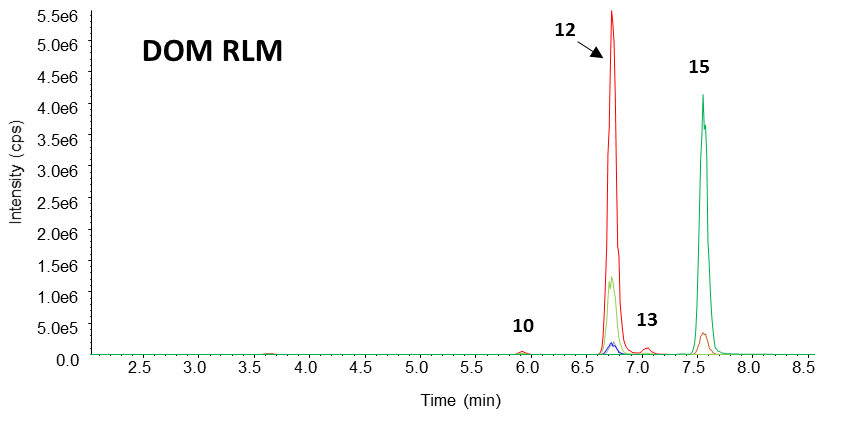
**

Figure S3: LC-MS/MS chromatogram of DOM and its glucuronides isolated after incubation of DOM with RLM. 10: unknown DOM-GlcAc b, 12: DOM-3-GlcAc, 13: DOM-15-GlcAc, 15: DOM.

**
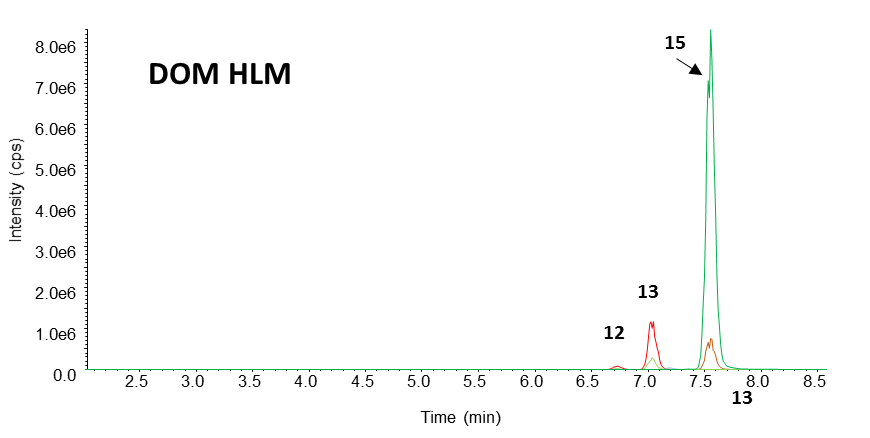
**

Figure S4: LC-MS/MS chromatogram of DOM and its glucuronides isolated after incubation of DOM with HLM. 12: DOM-3-GlcAc, 13: DOM-15-GlcAc, 15: DOM.

**HR-MS/MS spectra:**

DON-derived glucuronides:

Compound 1:


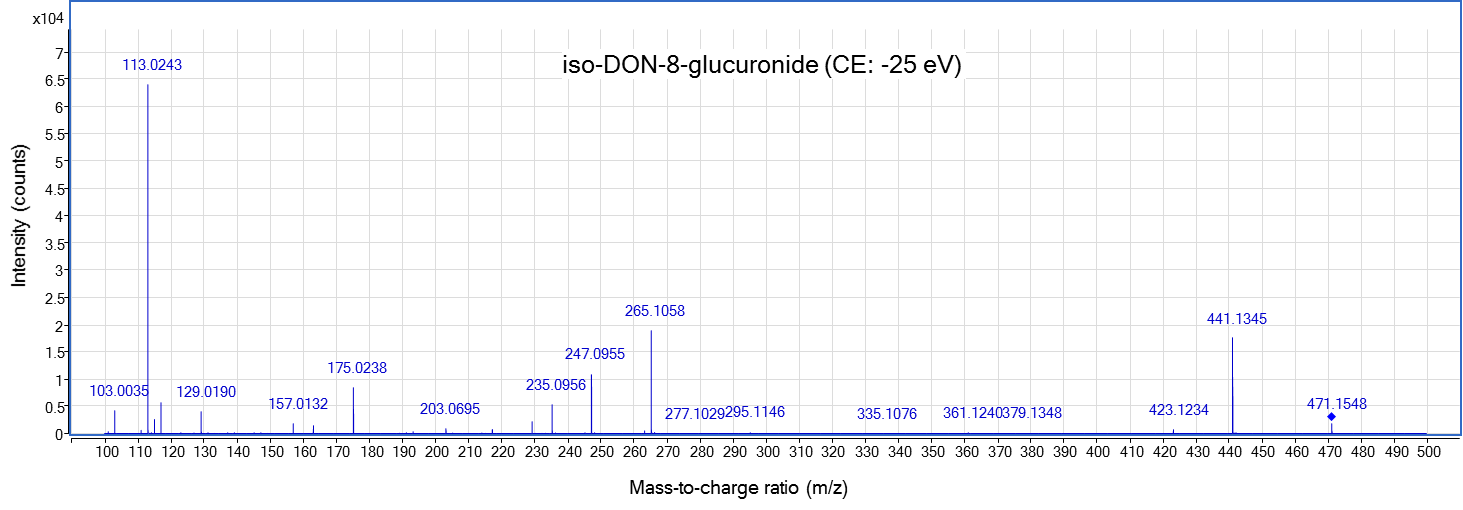


Compound 2:


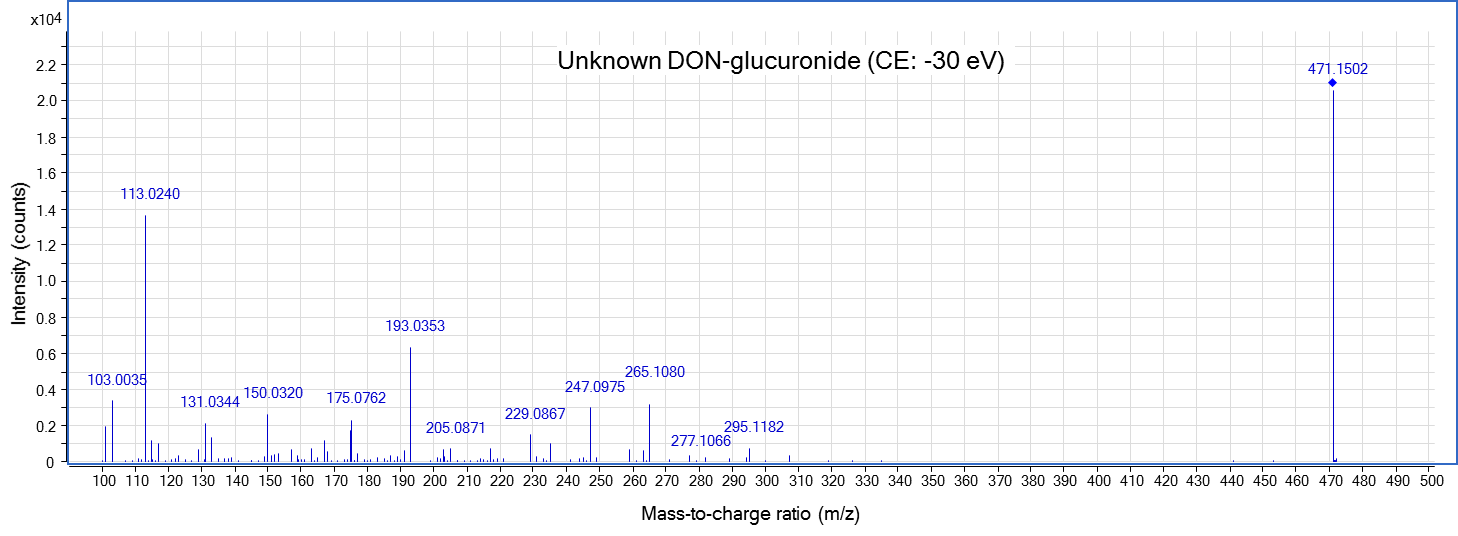


Compound 5:


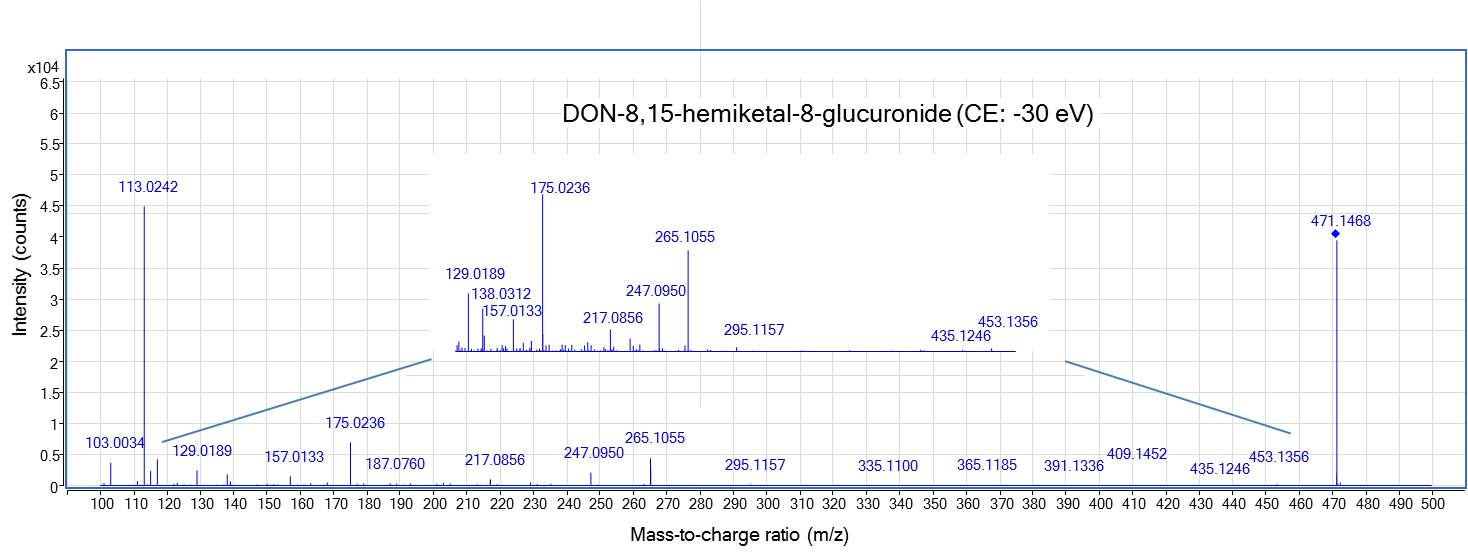


Compound 6:


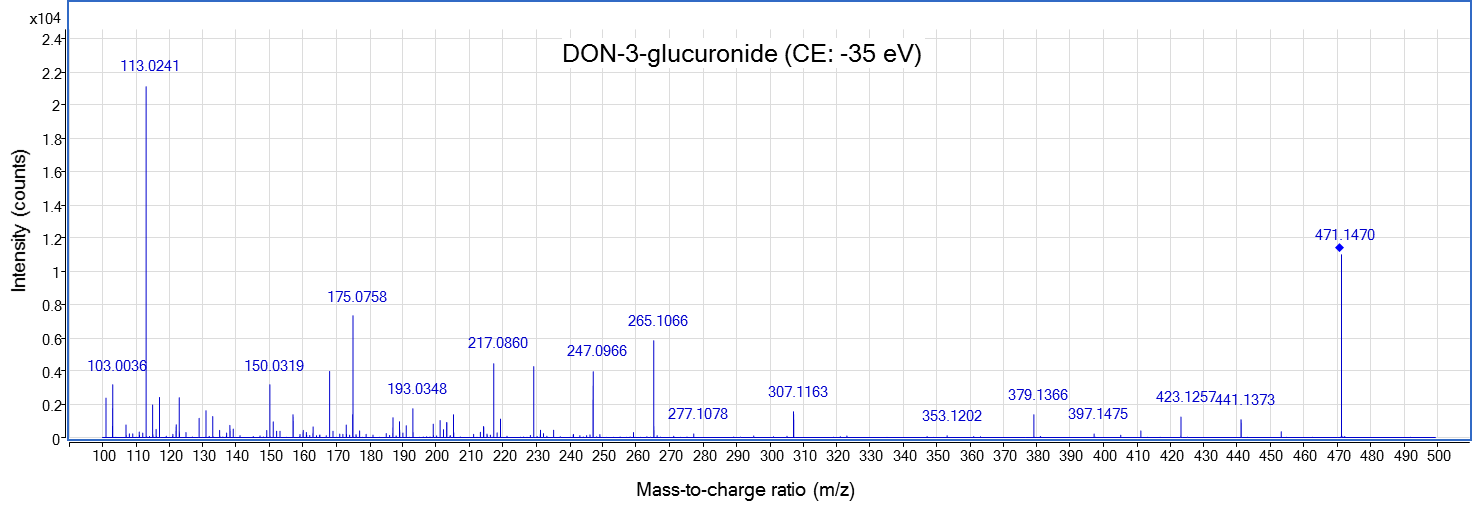


Compound 7:


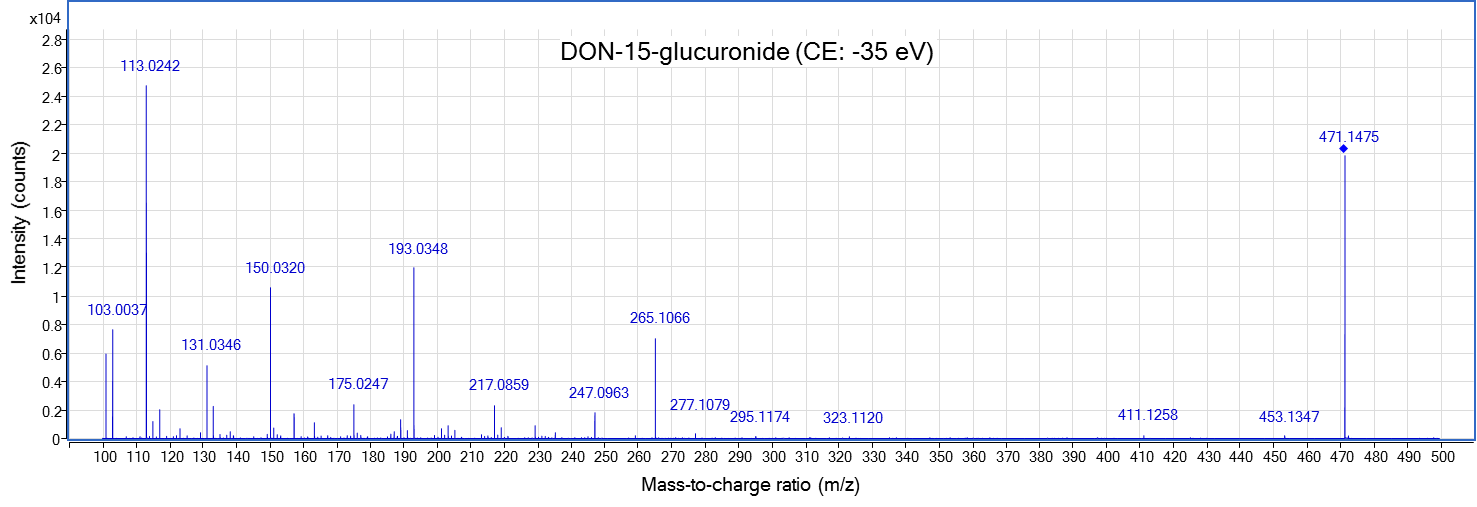


Compound 9:


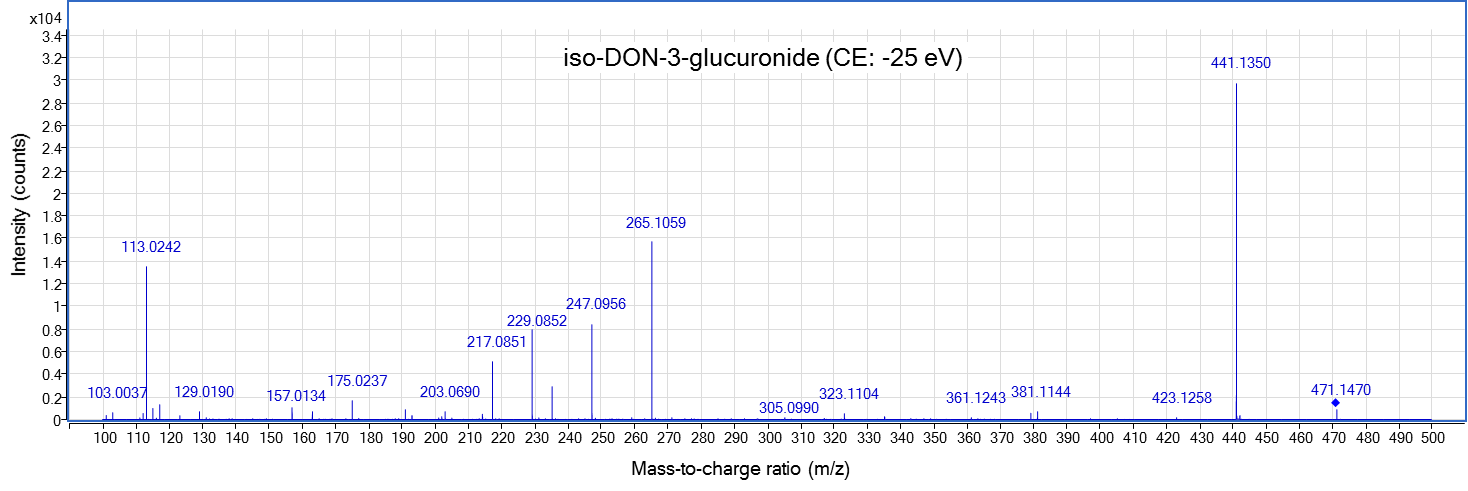


Additional compound produced upon incubation of iso-DON with RLM:


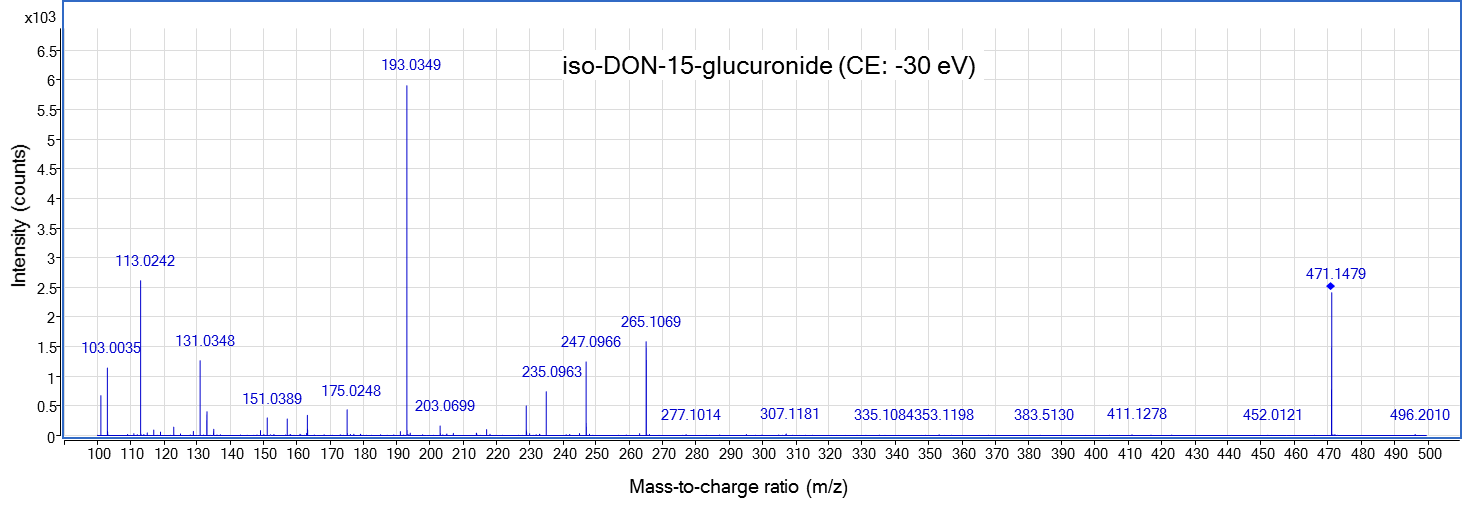


DOM-derived glucuronides:

Compound 3:


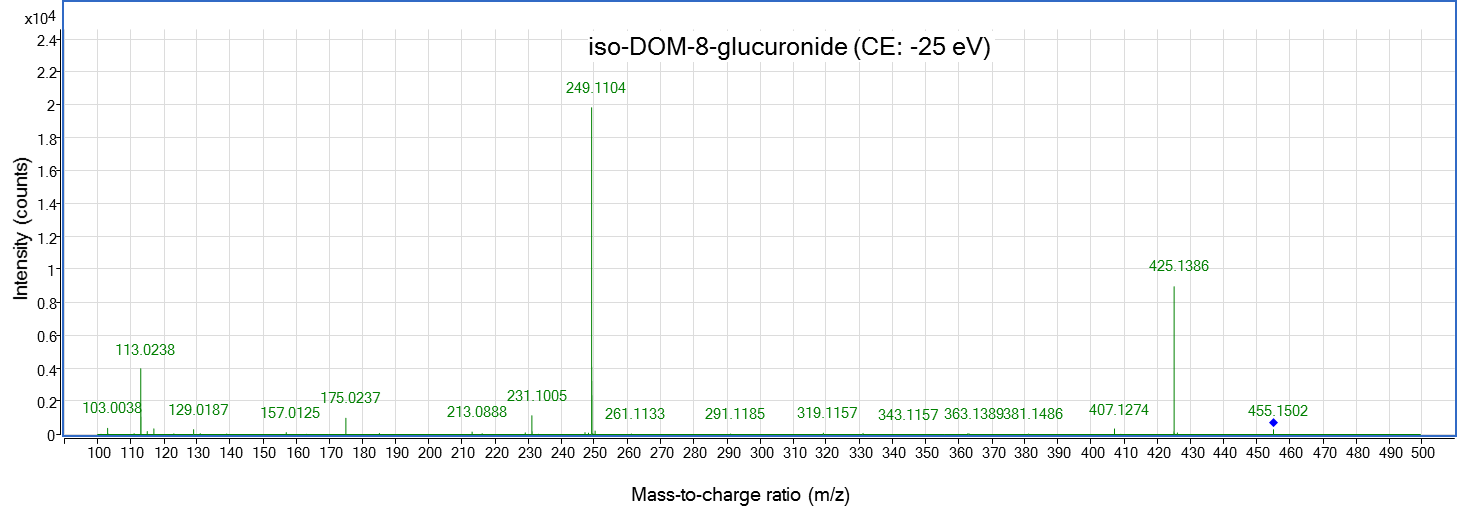


Compound 4:


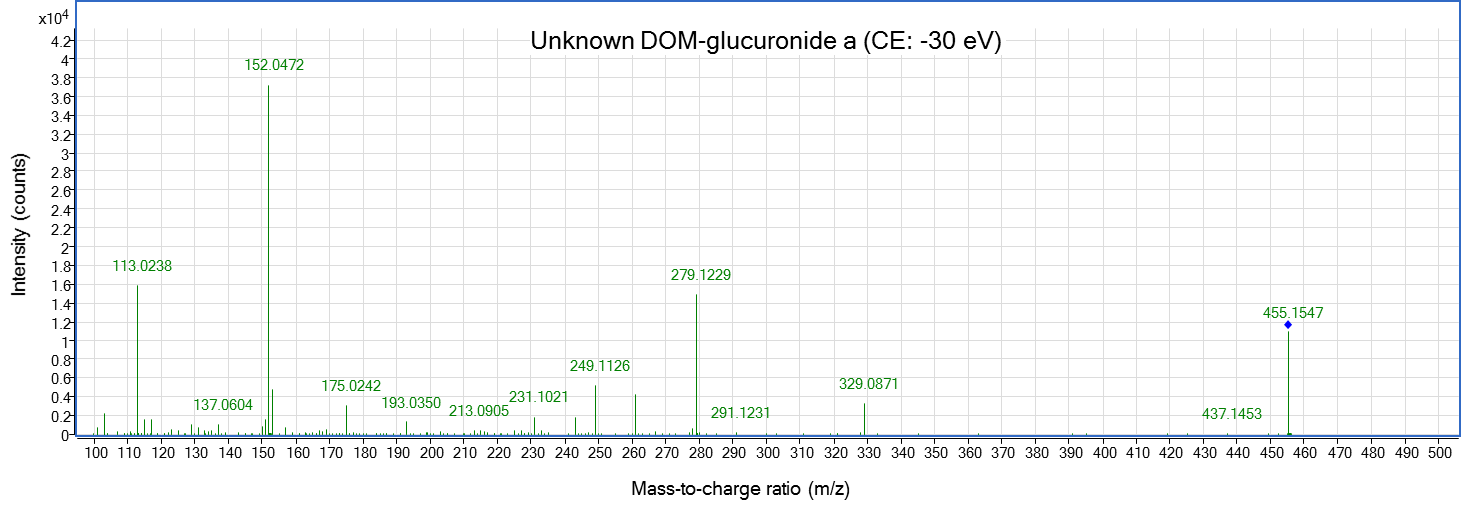


Compound 10:


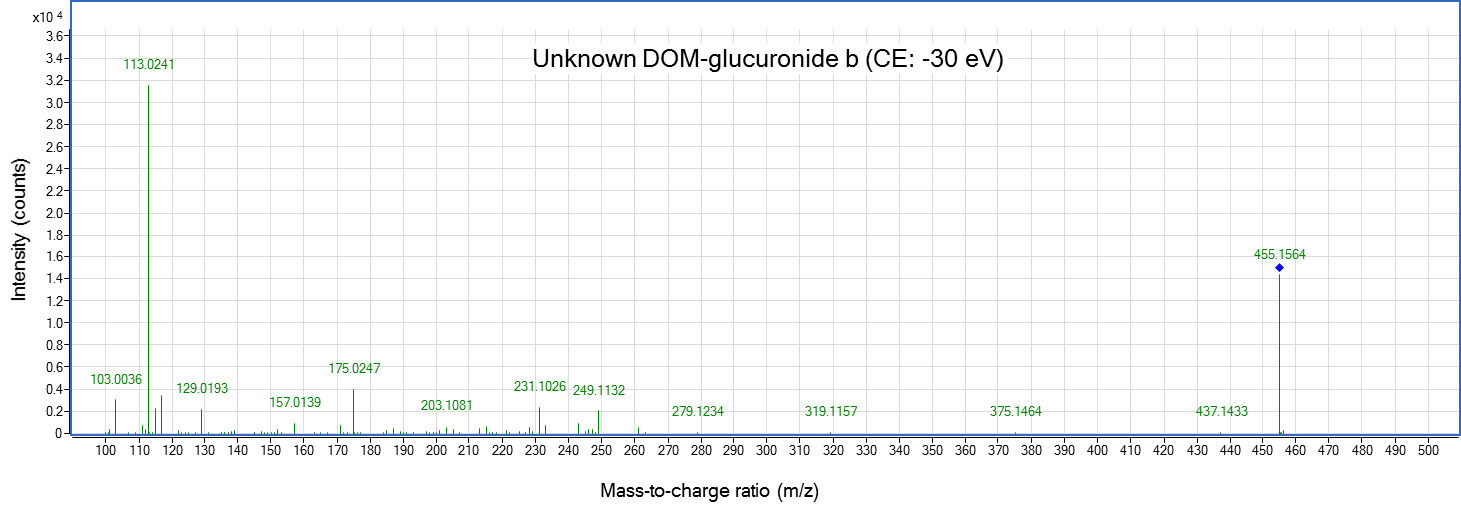


Compound 12:


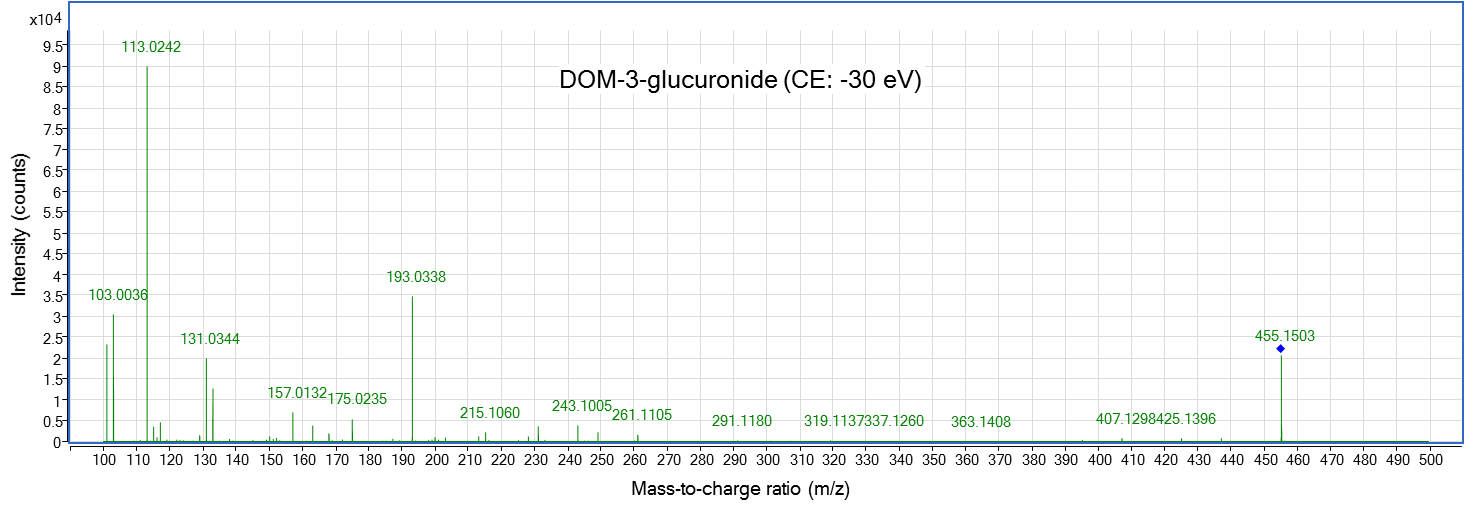


Compound 13:


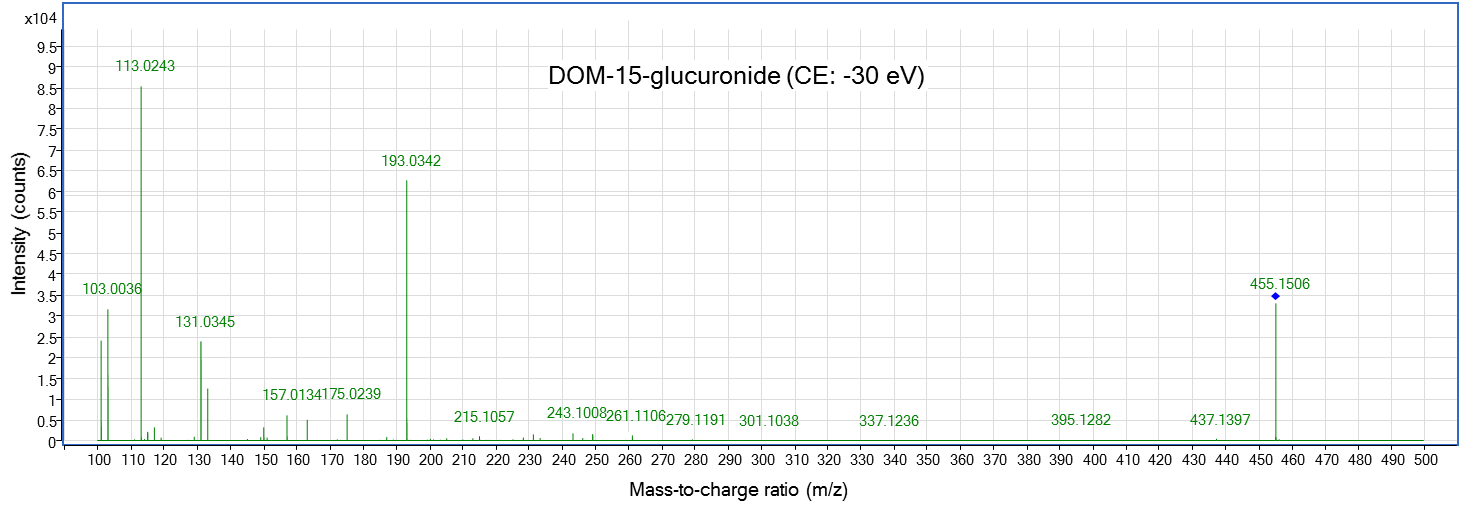


Compound 14:


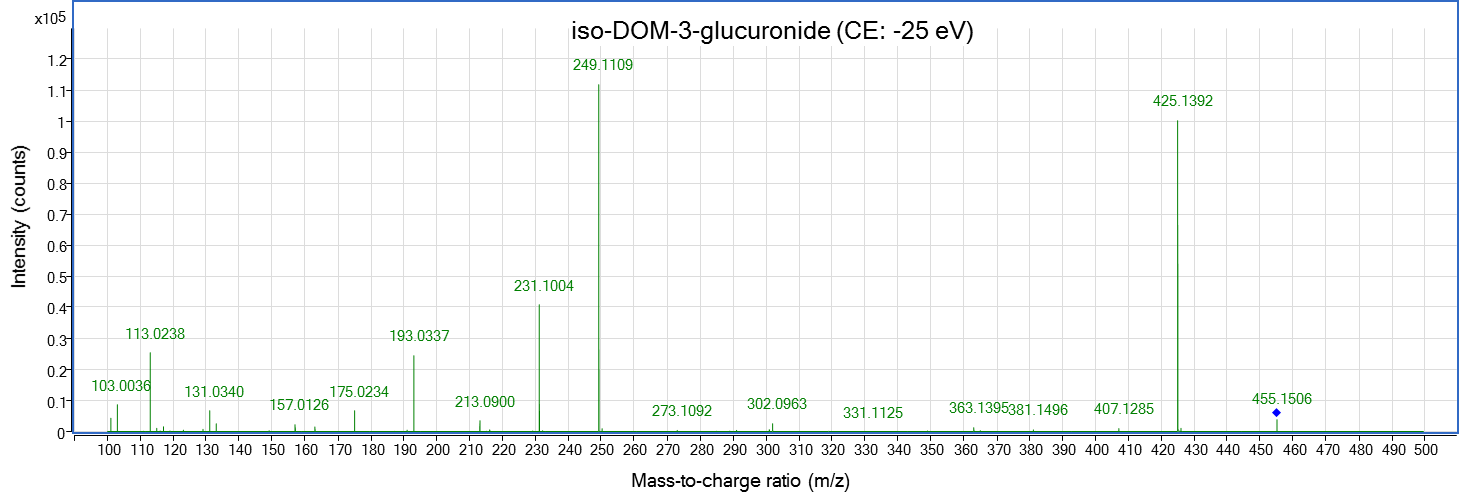


Additional compound produced upon incubation of iso-DOM with RLM:


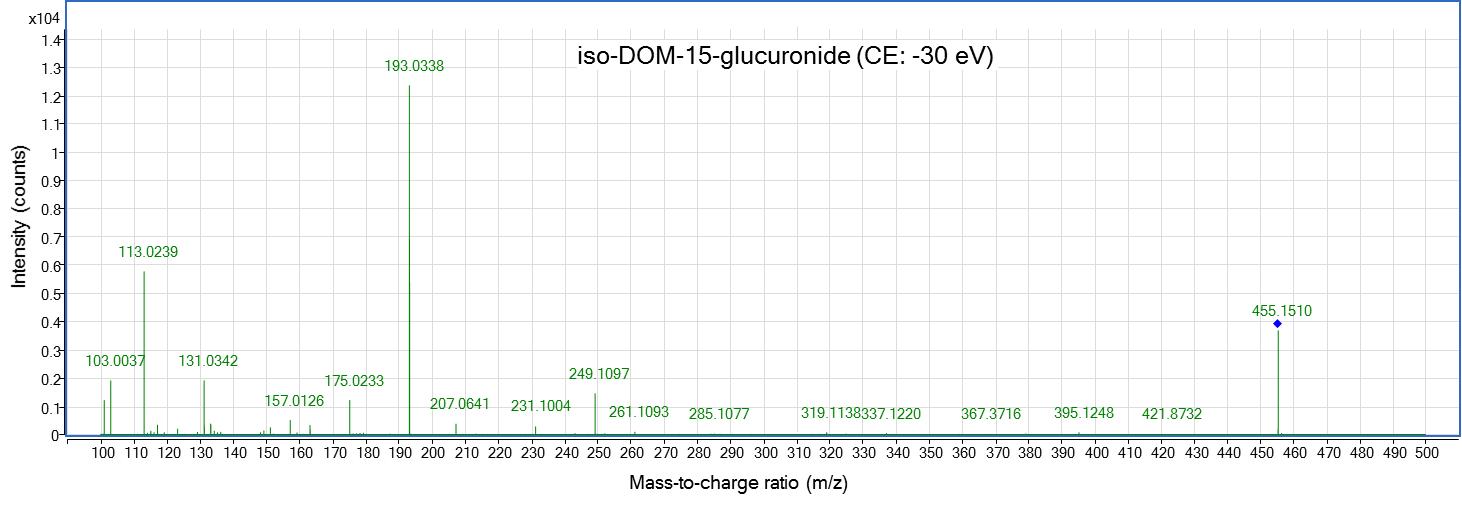


Parent compounds:

Compound 8:


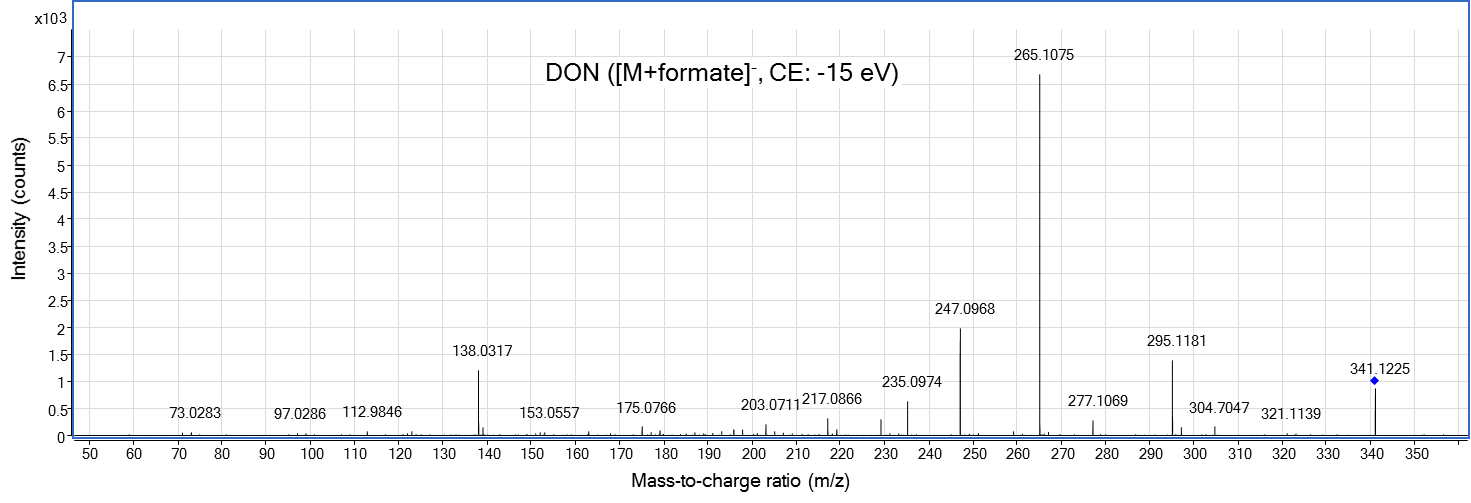


Compound 11:


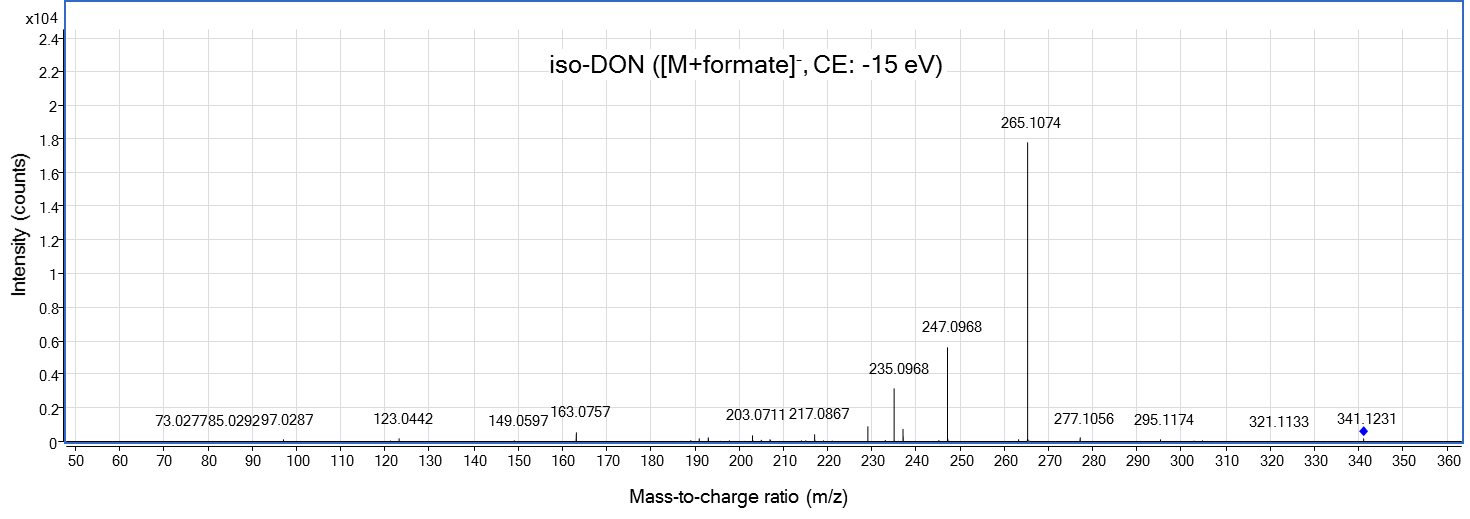


Compound 15:


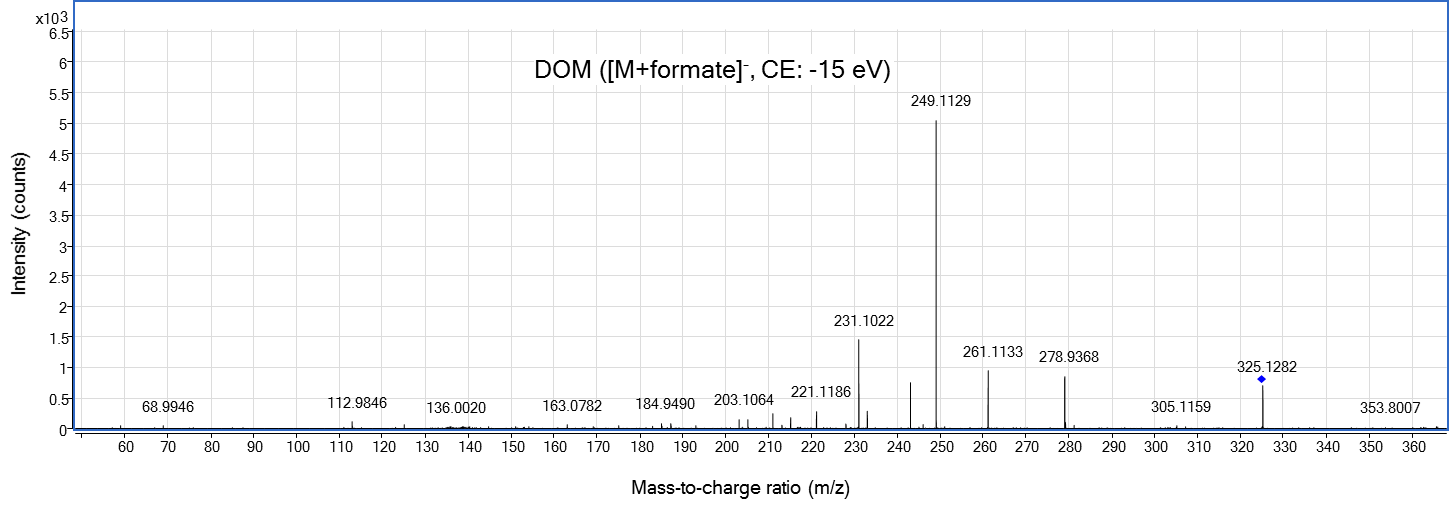


Compound 16:


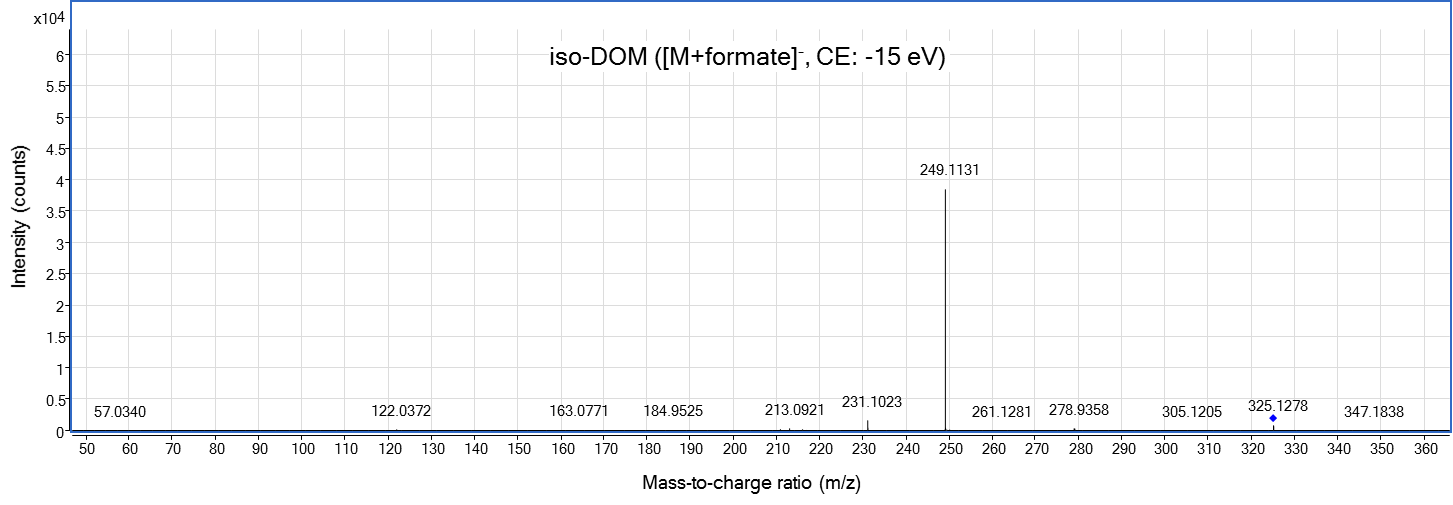


**NMR spectra:**

Iso-DON glucuronides:

Compound 1: iso-DON-8-glucuronide


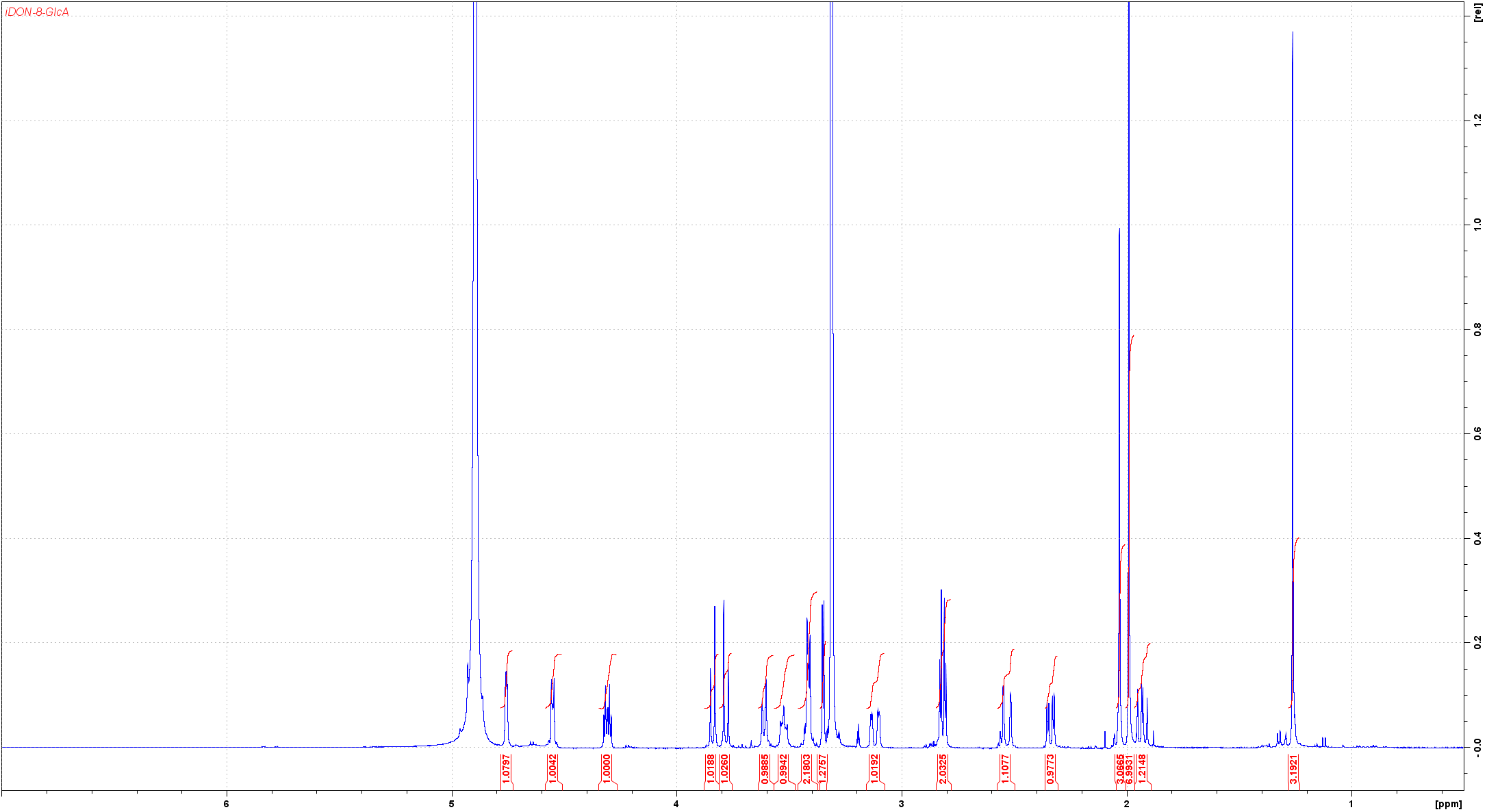


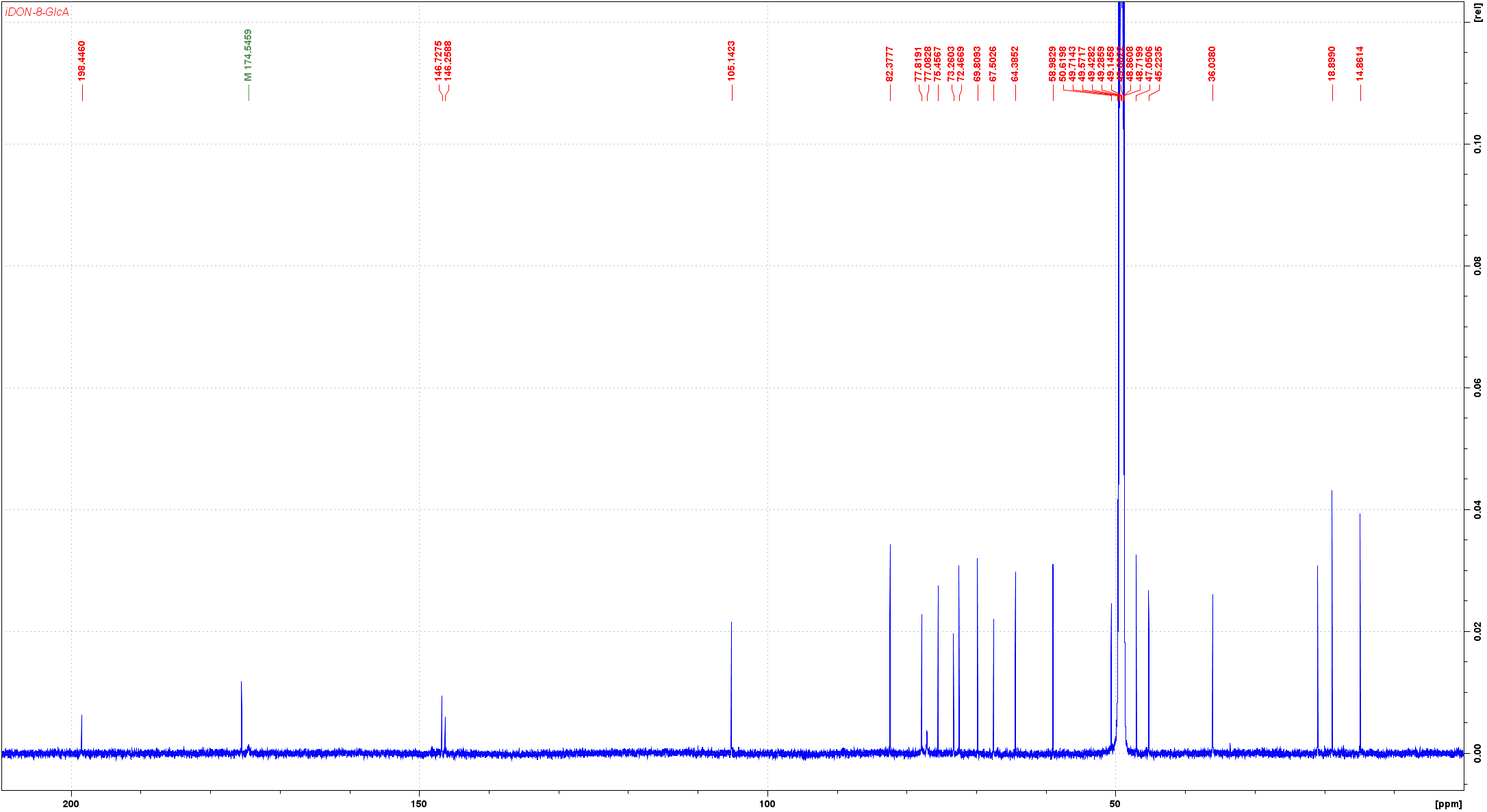


Compound 9: iso-DON-3-glucuronide


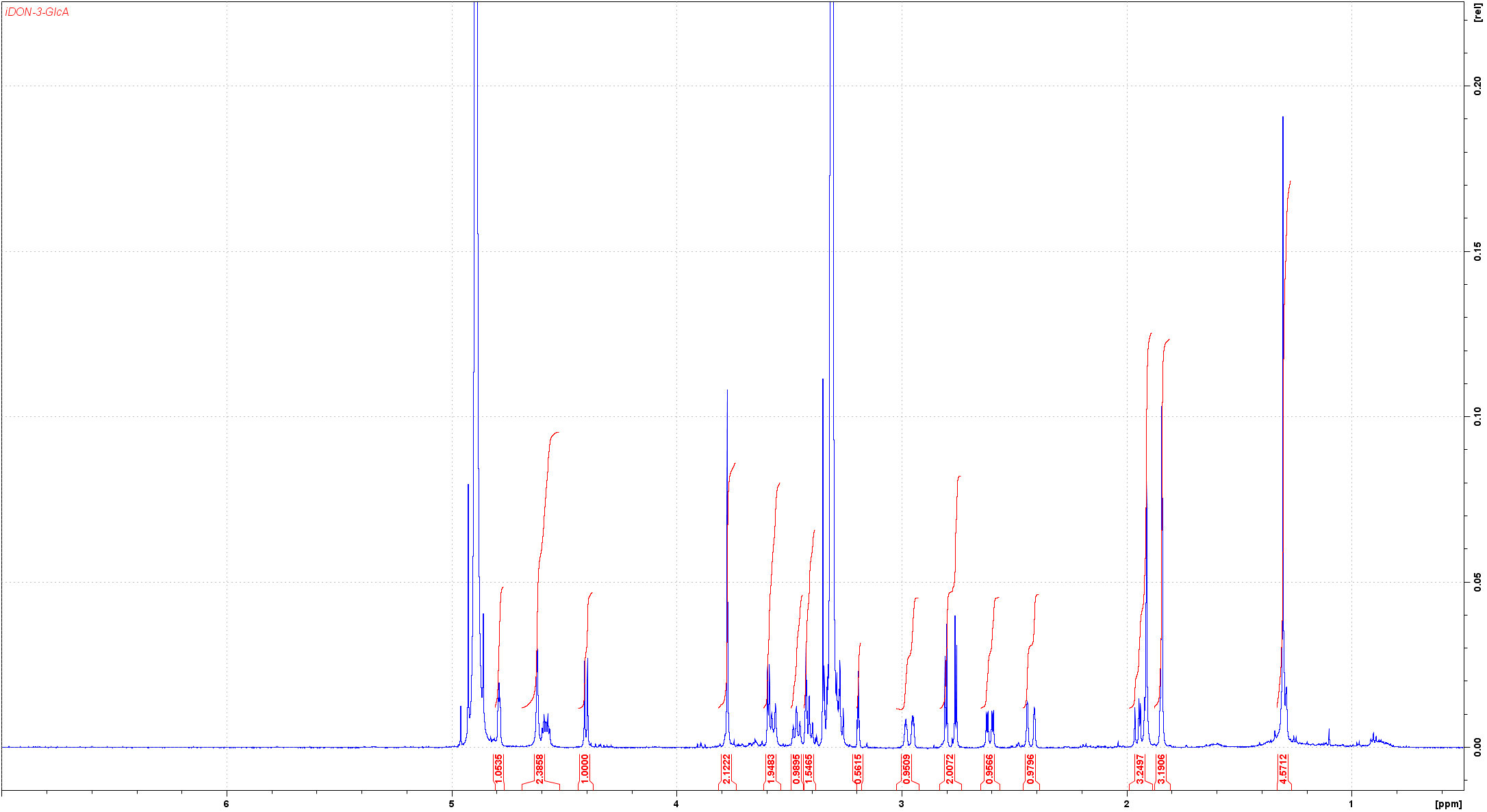


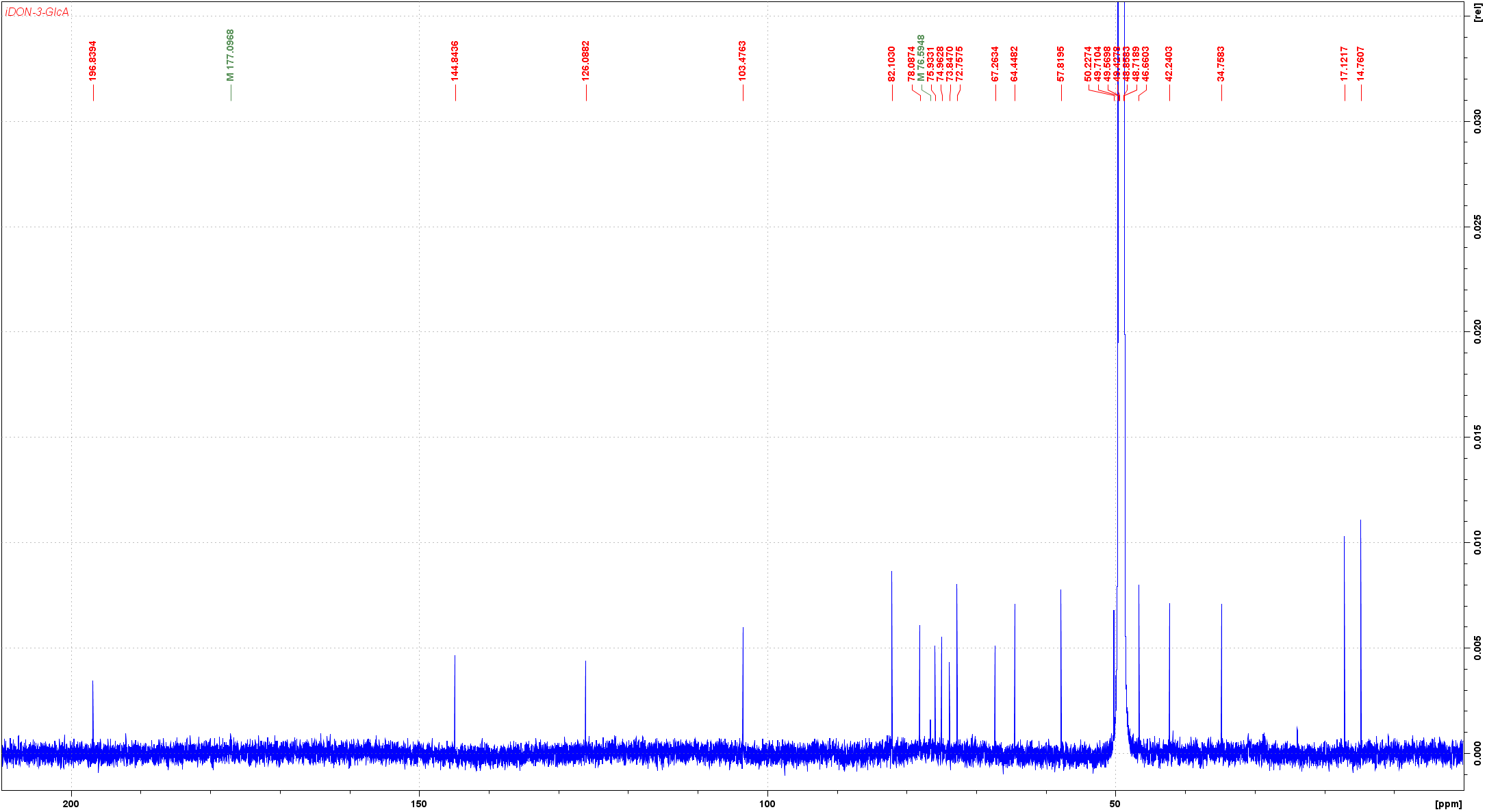


Iso-DOM glucuronides:

Compound 3: iso-DOM-8-glucuronide


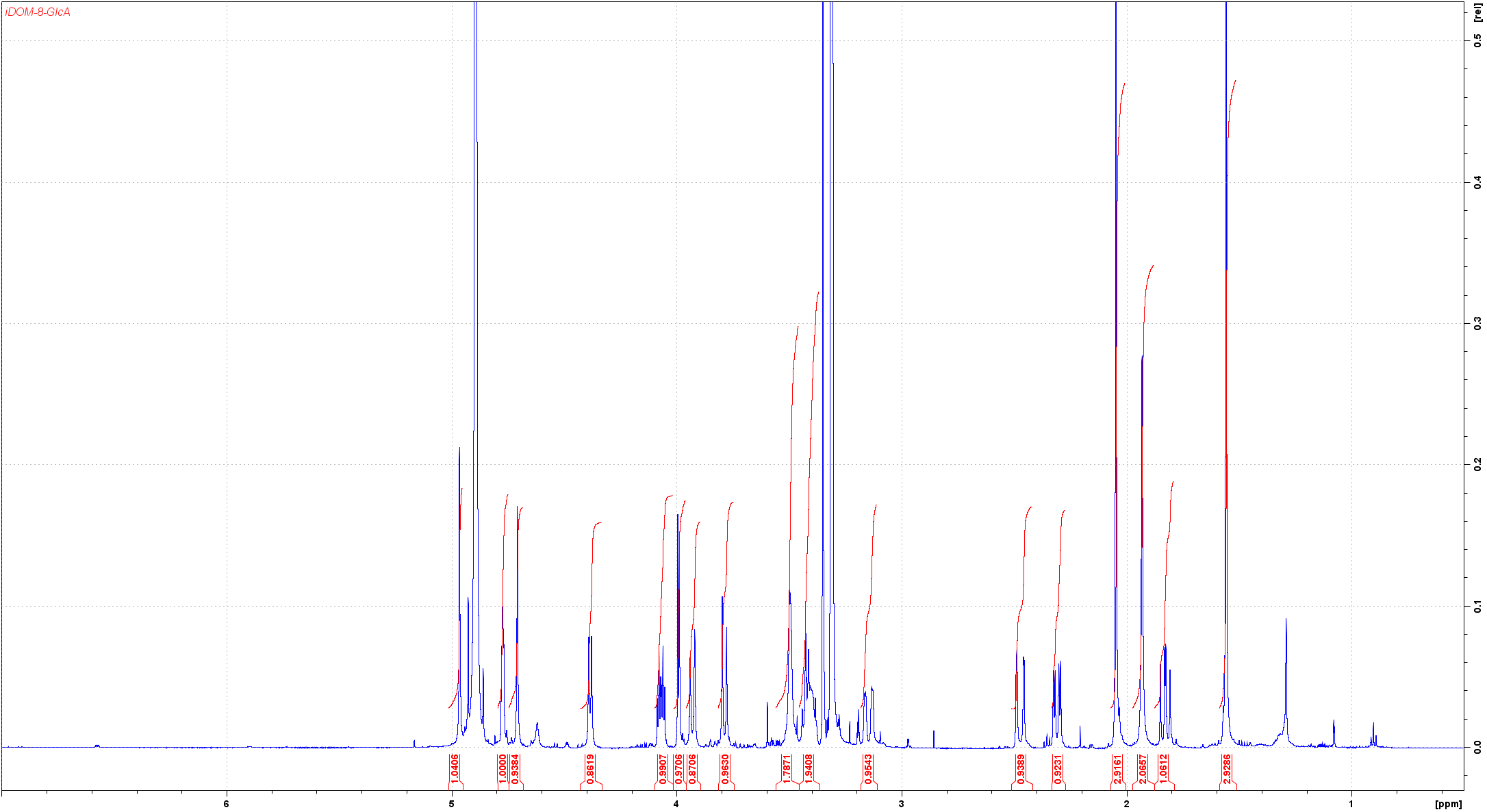


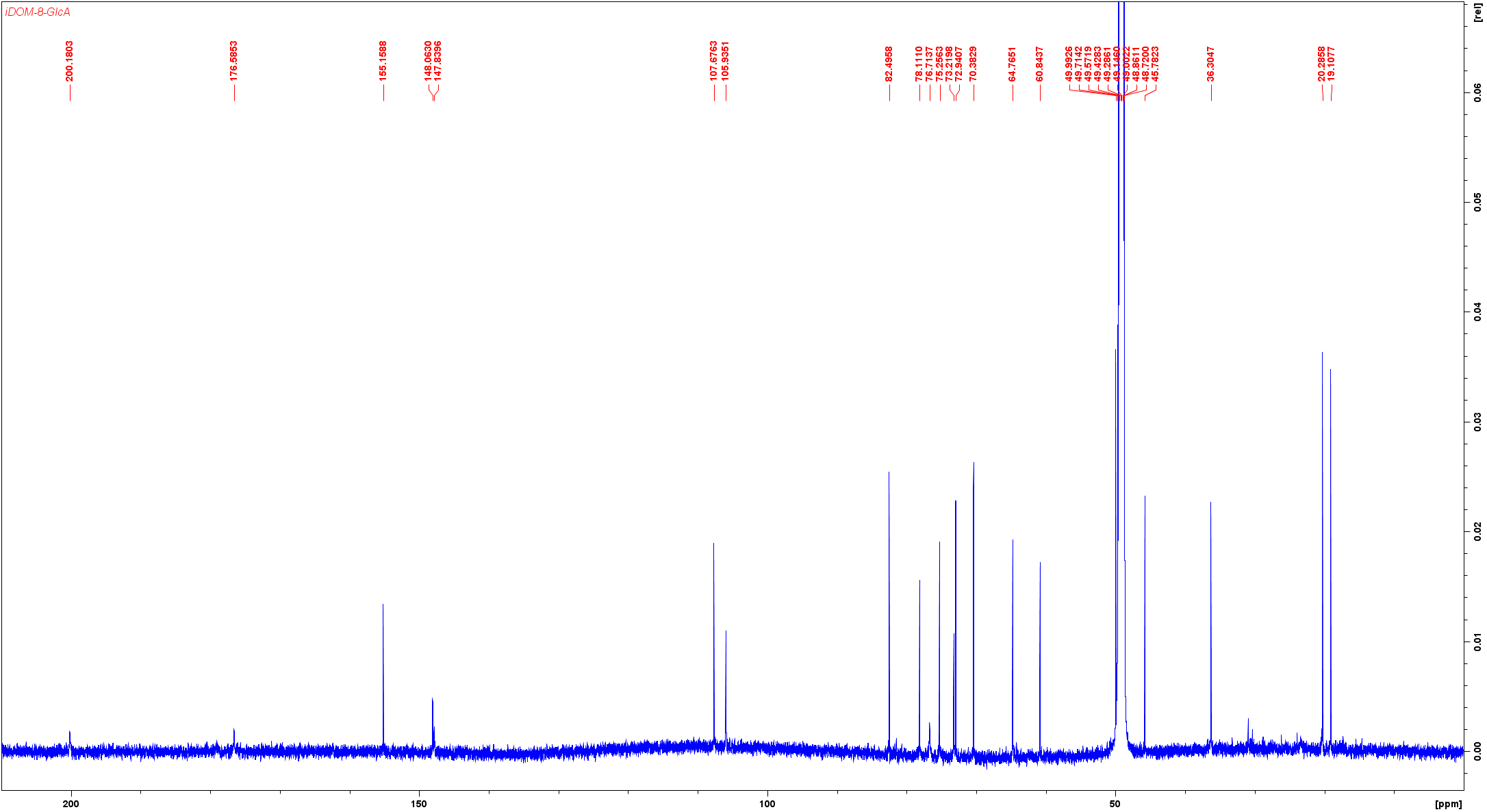


Compound 14: iso-DOM-3-glucuronide


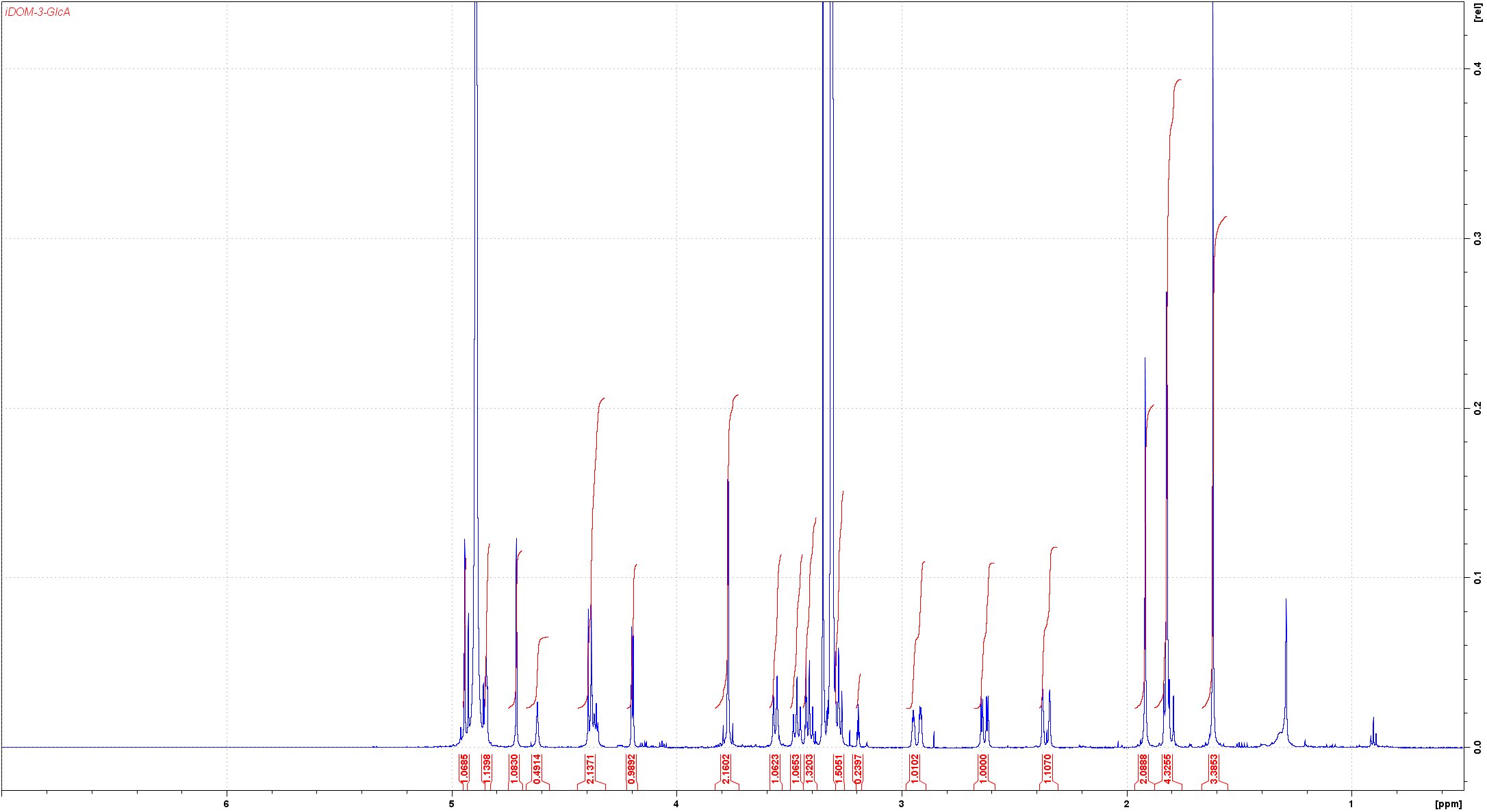


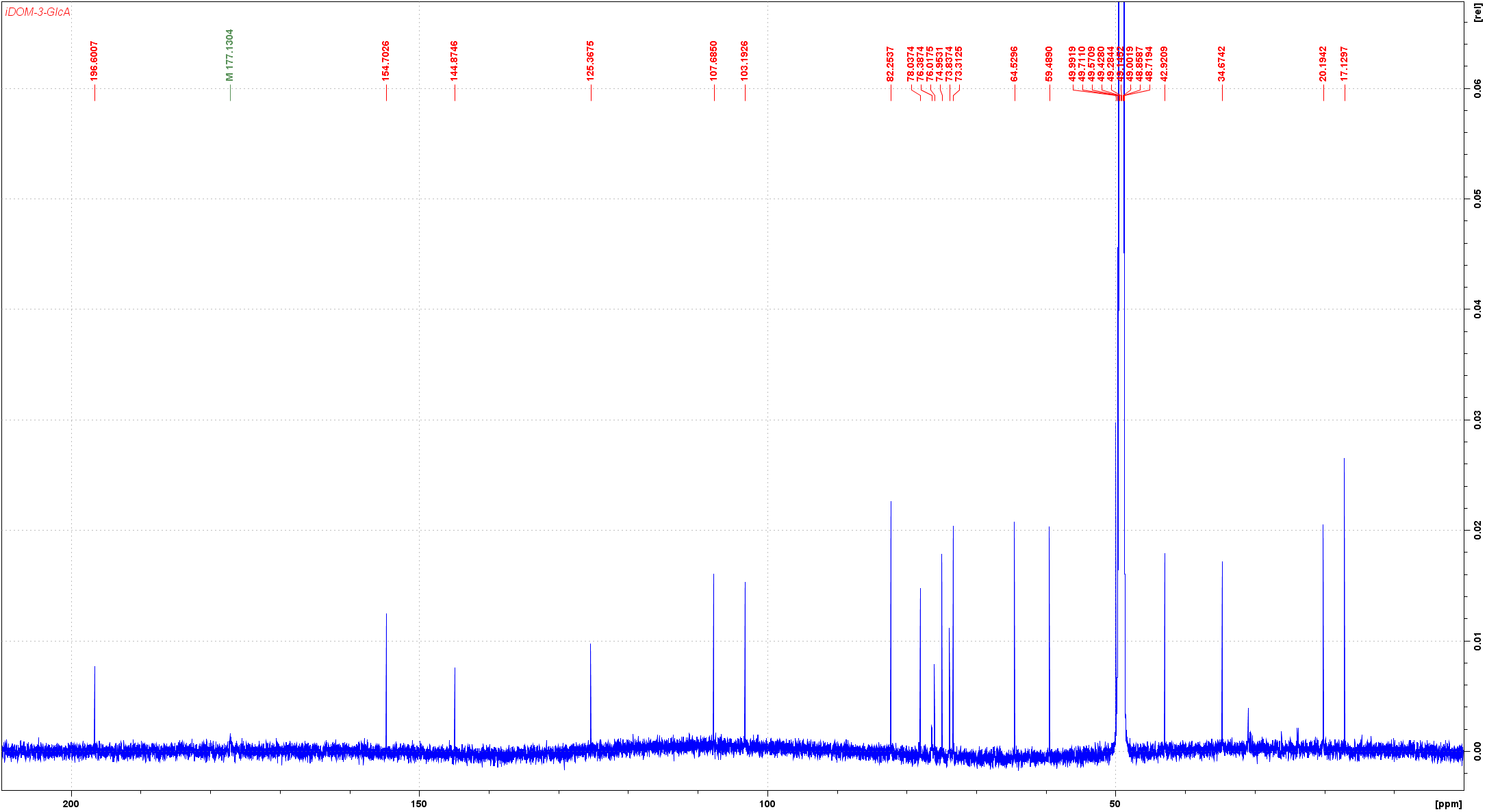


Parent compounds:

Compound 11: iso-DON


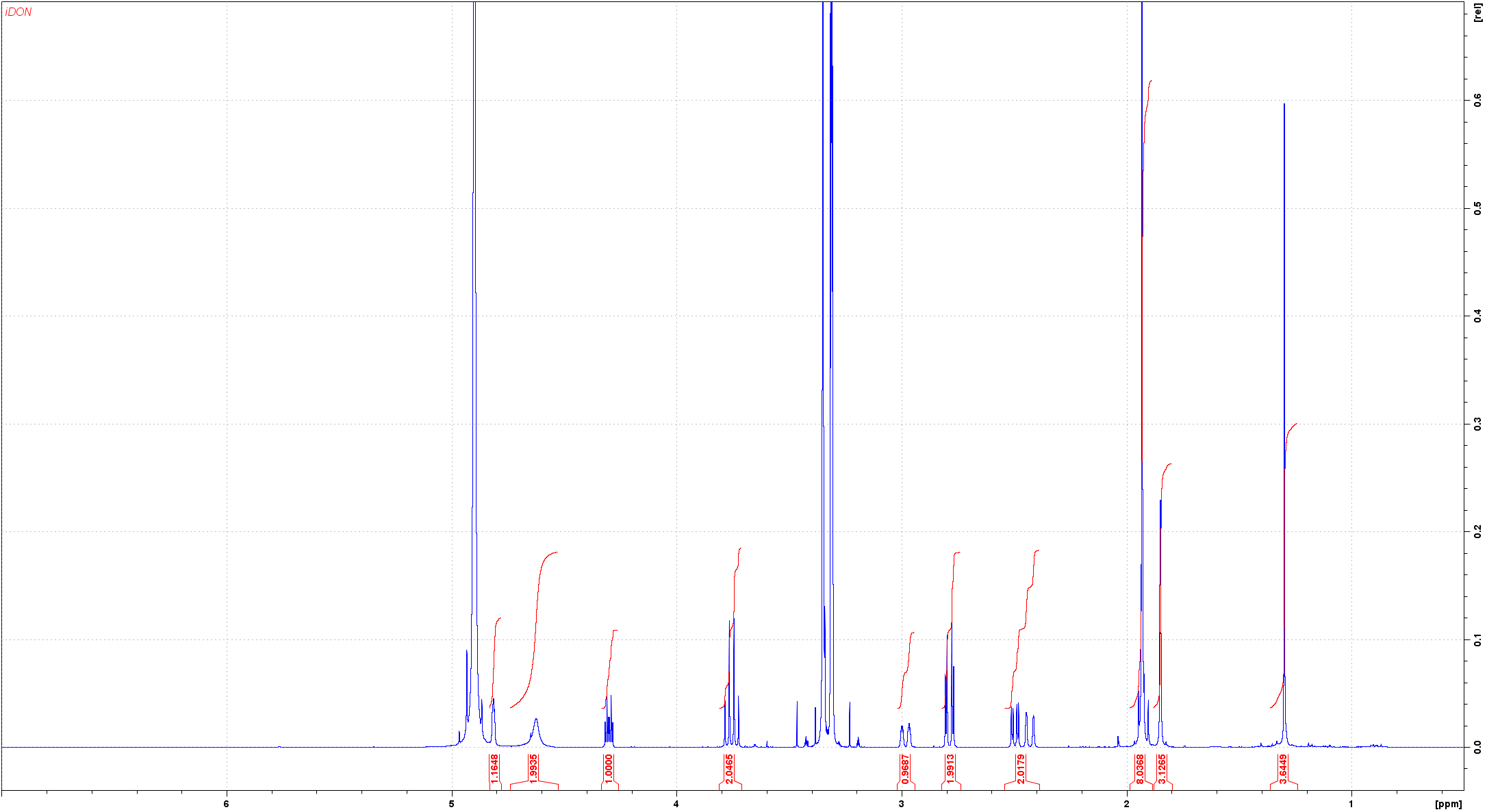


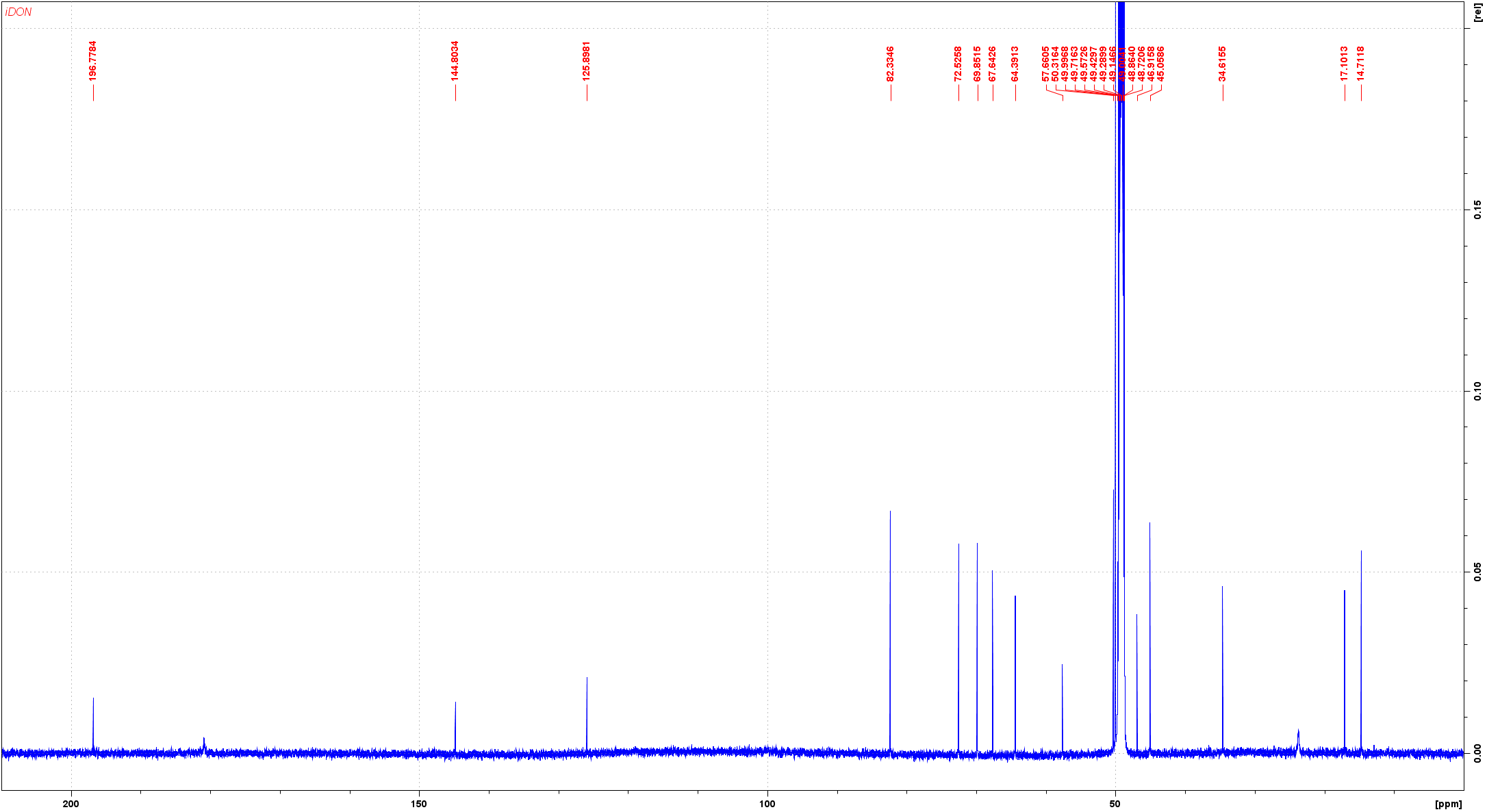


Compound 16: iso-DOM


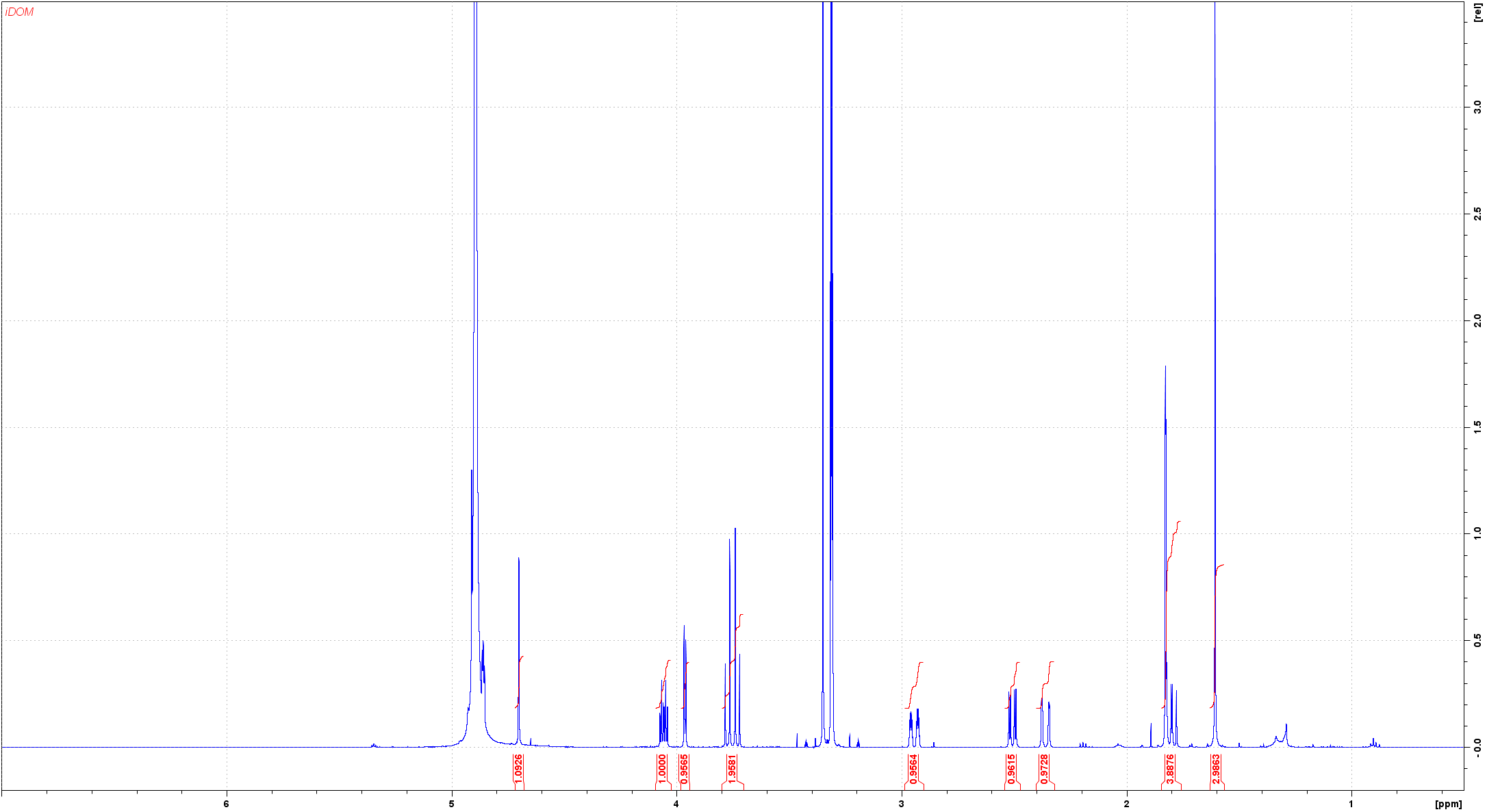


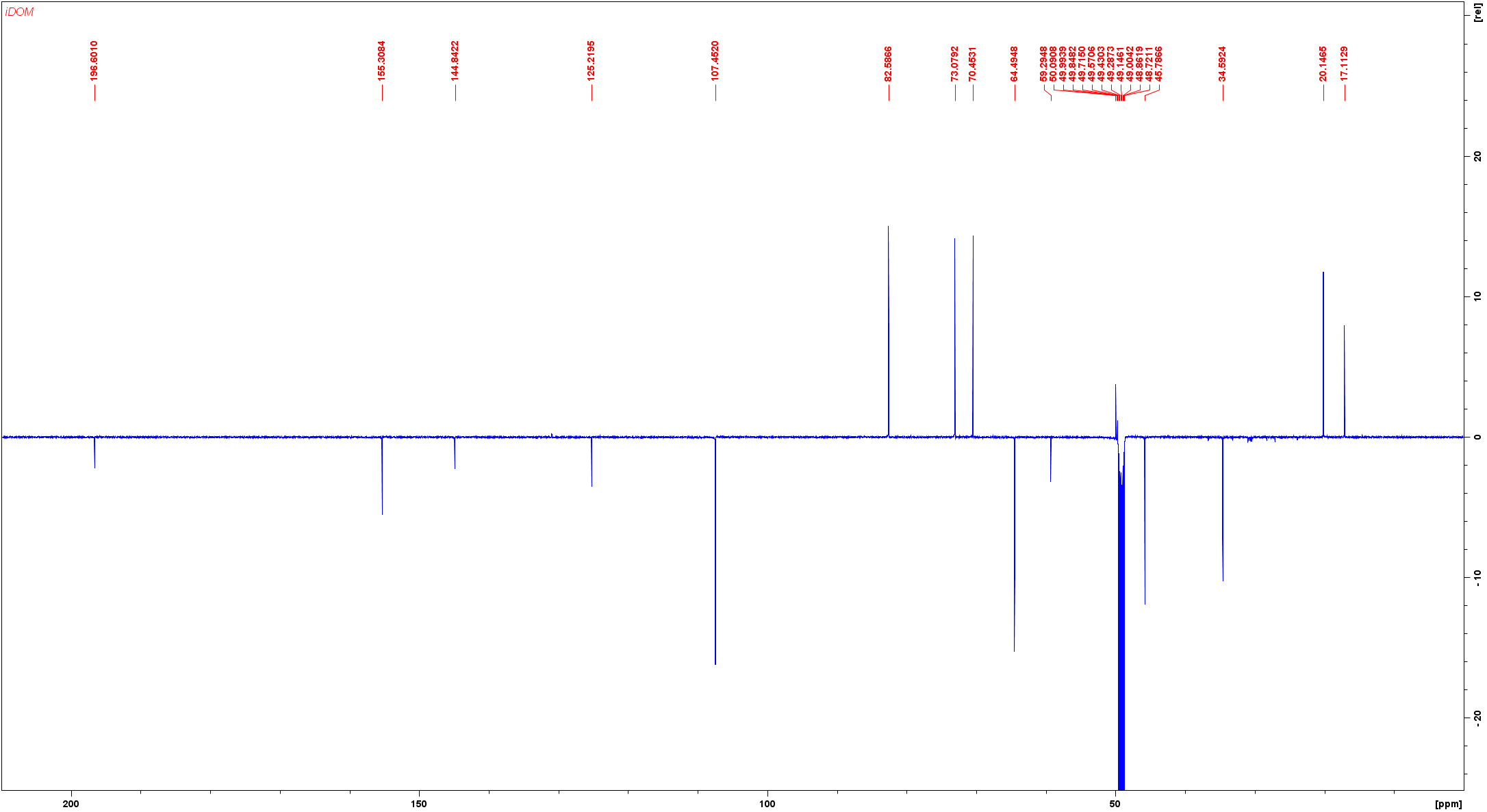


DOM glucuronides:

Compound 12: DOM-3-glucuronide


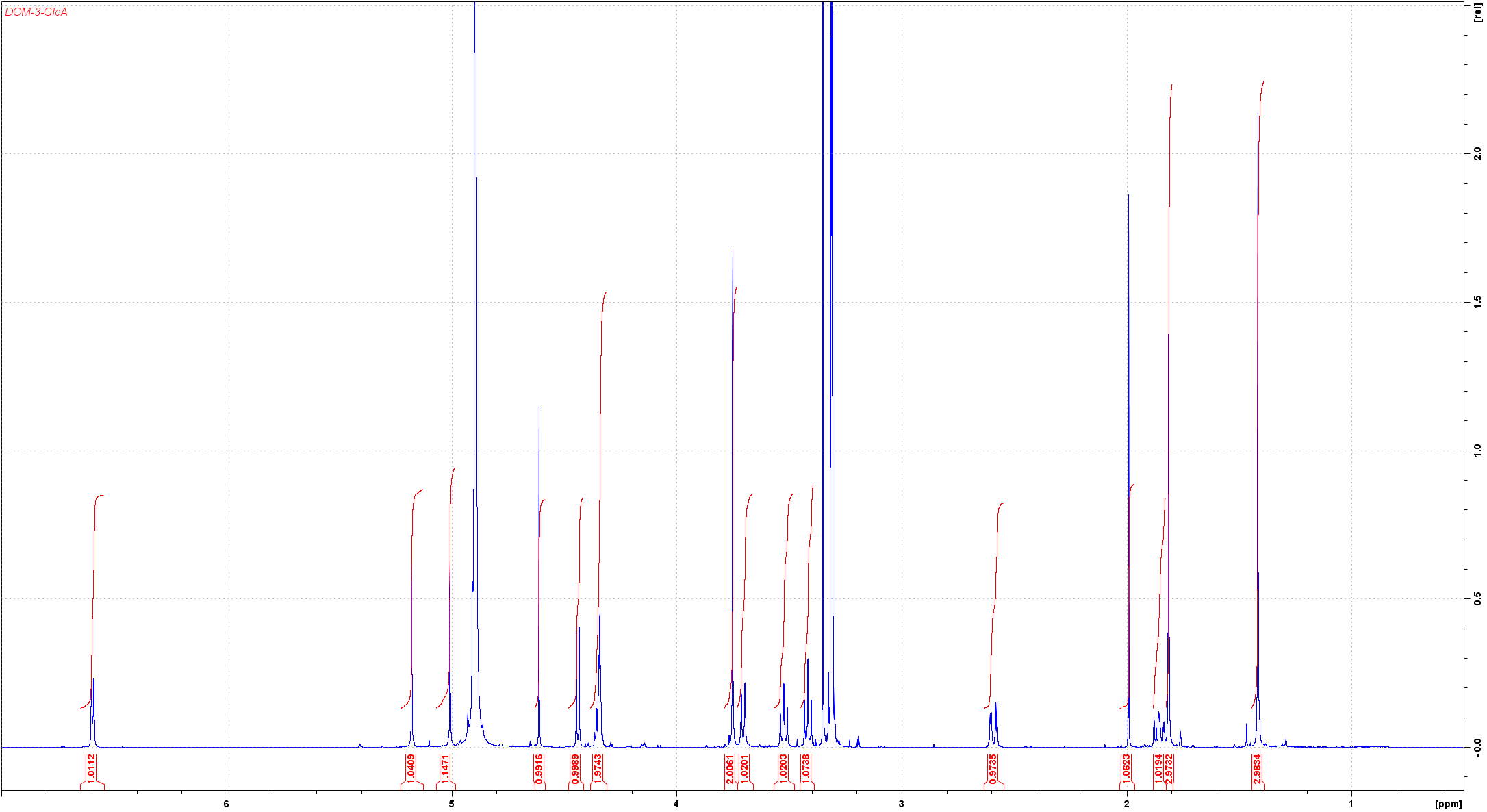


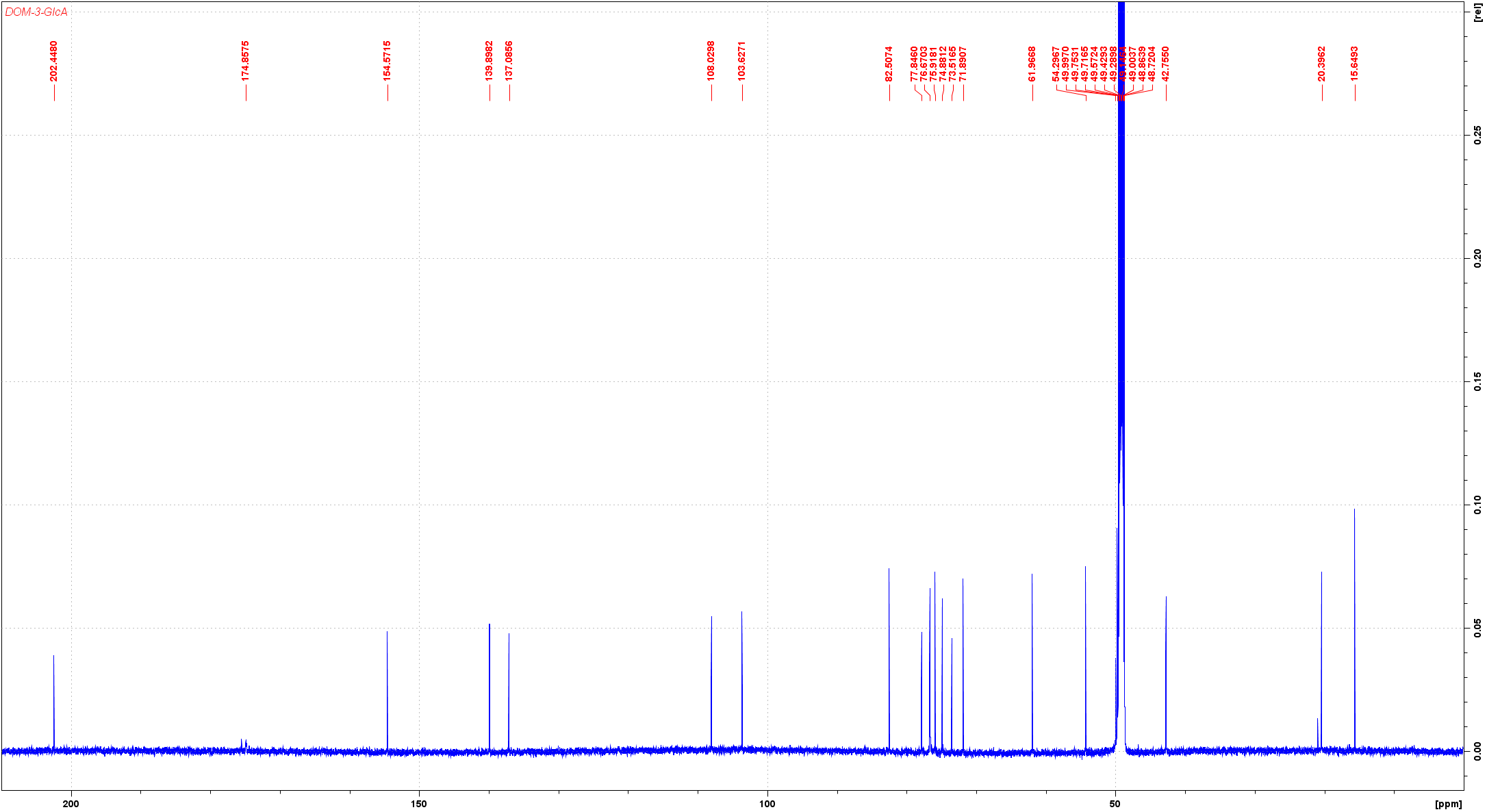


Compound 13: DOM-15-glucuronide


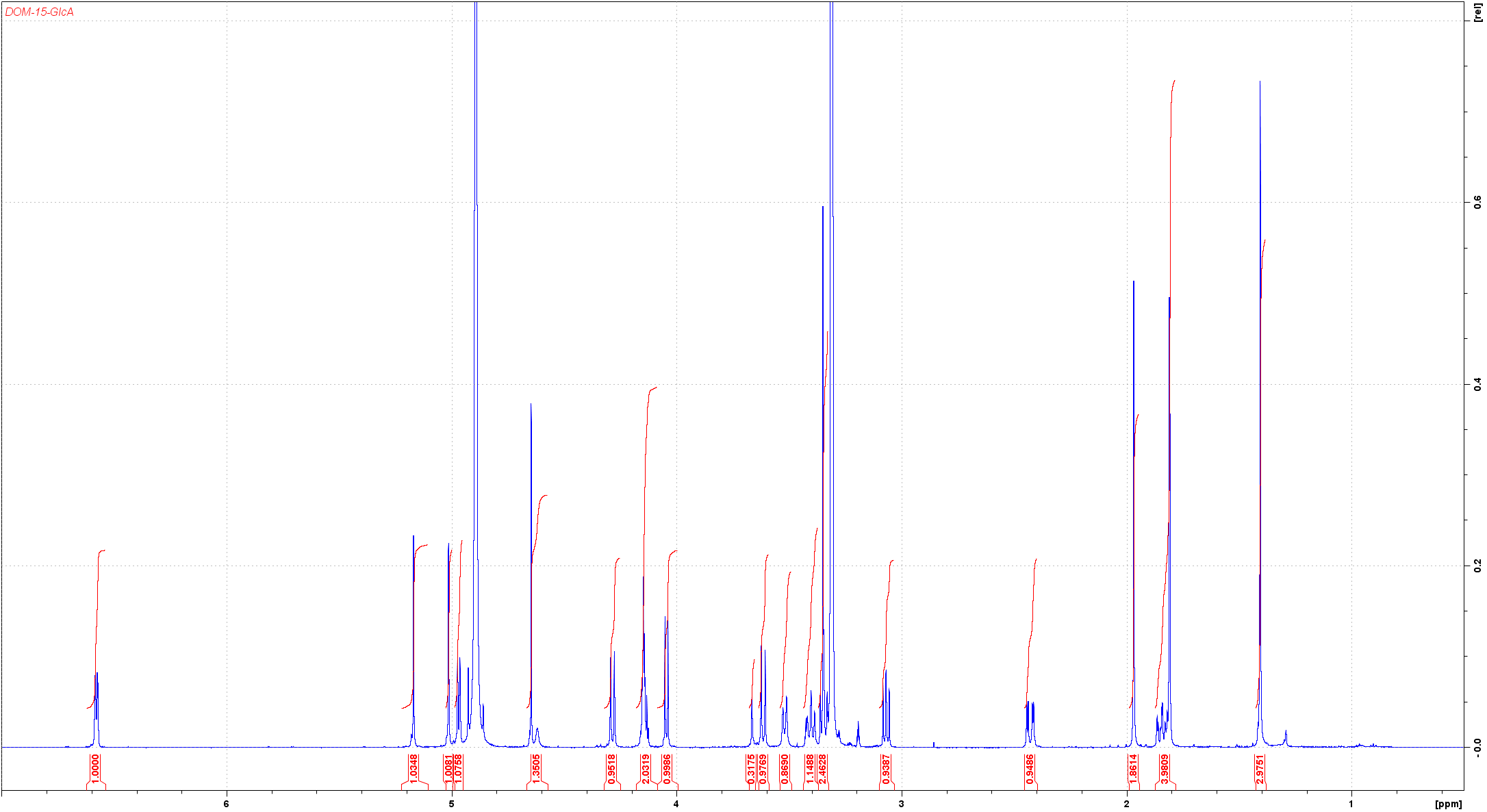


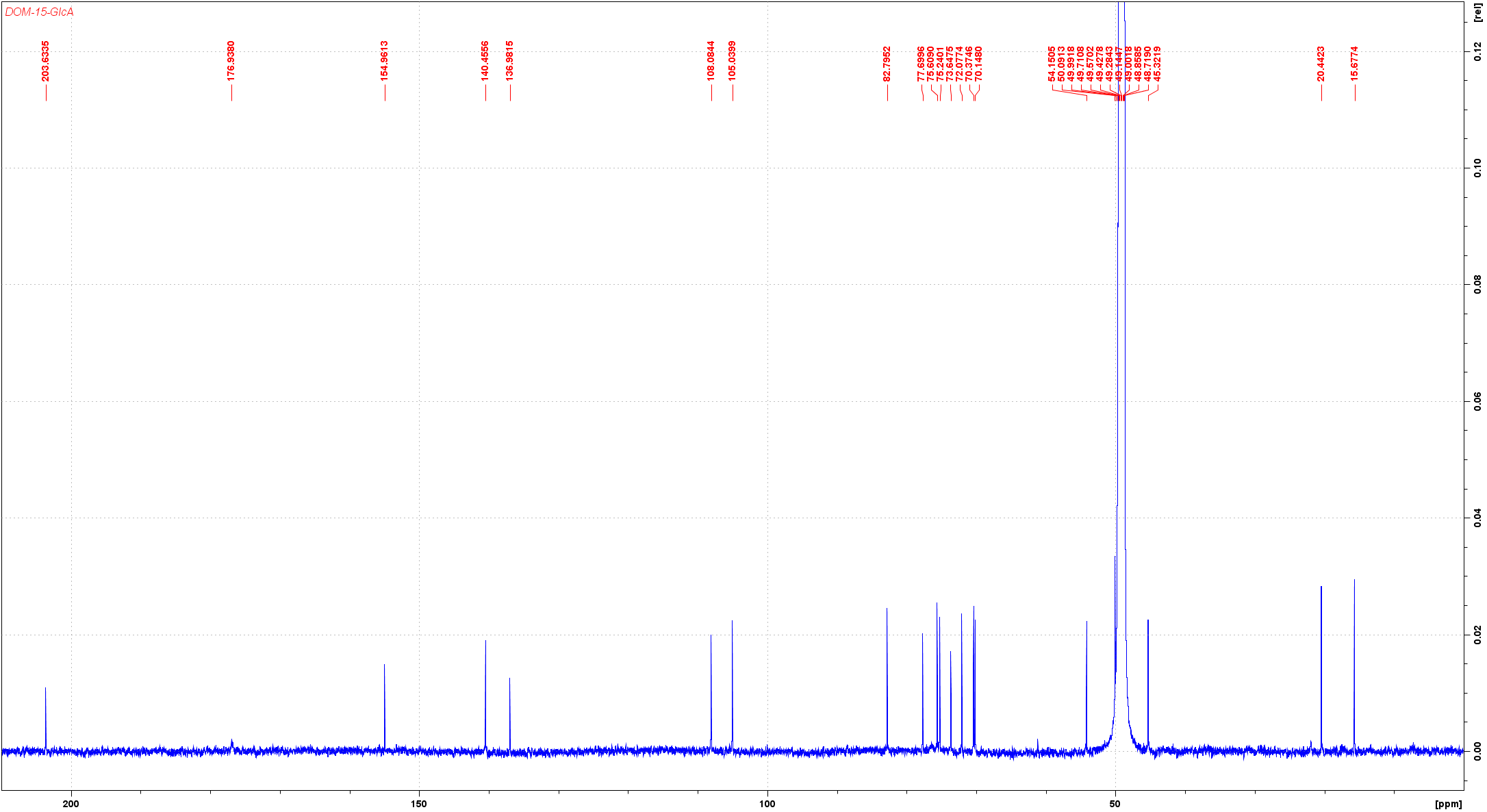

Supplement: Supplementary file 1 — Supplementary material 1 (DOCX 2039 kb) [file 204_2017_2012_MOESM1_ESM.docx]
